# Structural Similarities between Brain and Linguistic Data Provide Evidence of Semantic Relations in the Brain

**DOI:** 10.1371/journal.pone.0065366

**Published:** 2013-06-14

**Authors:** Colleen E. Crangle, Marcos Perreau-Guimaraes, Patrick Suppes

**Affiliations:** 1 School of Computing and Communications, Lancaster University, Lancaster, United Kingdom; 2 Converspeech LLC, Palo Alto, California, United States of America; 3 Suppes Brain Lab, Center for the Study of Language and Information, Stanford University, Stanford, California, United States of America; University of Leicester, United Kingdom

## Abstract

This paper presents a new method of analysis by which structural similarities between brain data and linguistic data can be assessed at the semantic level. It shows how to measure the strength of these structural similarities and so determine the relatively better fit of the brain data with one semantic model over another. The first model is derived from WordNet, a lexical database of English compiled by language experts. The second is given by the corpus-based statistical technique of latent semantic analysis (LSA), which detects relations between words that are latent or hidden in text. The brain data are drawn from experiments in which statements about the geography of Europe were presented auditorily to participants who were asked to determine their truth or falsity while electroencephalographic (EEG) recordings were made. The theoretical framework for the analysis of the brain and semantic data derives from axiomatizations of theories such as the theory of differences in utility preference. Using brain-data samples from individual trials time-locked to the presentation of each word, ordinal relations of similarity differences are computed for the brain data and for the linguistic data. In each case those relations that are invariant with respect to the brain and linguistic data, and are correlated with sufficient statistical strength, amount to structural similarities between the brain and linguistic data. Results show that many more statistically significant structural similarities can be found between the brain data and the WordNet-derived data than the LSA-derived data. The work reported here is placed within the context of other recent studies of semantics and the brain. The main contribution of this paper is the new method it presents for the study of semantics and the brain and the focus it permits on networks of relations detected in brain data and represented by a semantic model.

## Introduction

This paper presents a new method of analysis that assesses the extent to which brain data are structurally similar to linguistic data. It allows us to evaluate the relatively better fit of the brain data with one semantic model over another. The first model is derived from WordNet, a lexical database of English compiled by experts in which words are grouped into sets of cognitive synonyms called synsets, each set expressing a distinct concept, with relations such as synonymy being defined between the synsets [1], [2]. The second model is given by a statistical technique for extracting from large document collections a measure of how similar two words are to each other in terms of how similar their contexts of use are within those documents [3], [4]. Such a statistical method is said to reveal semantic relations latent in the texts and is referred to by the acronym LSA (latent semantic analysis). The brain data are drawn from experiments in which statements about the geography of Europe were presented auditorily to participants who were asked to determine their truth or falsity while electroencephalographic (EEG) recordings were made [5].

The theoretical framework for the analysis of the EEG data and the semantic data derives from the axiomatization of theories such as the theory of differences in utility preference or the theory of differences in psychological intensity. Using brain data samples from individual trials time-locked to the presentation of each word and extracted from the EEG recordings, ordinal relations of similarity differences are computed for the brain data and for linguistic data derived from the two models of semantic similarity. In each case those relations that are invariant with respect to the brain data and the linguistic data, and are correlated with sufficient statistical strength, amount to structural similarities between the brain and linguistic data. Our results show that many more statistically significant structural similarities can be found between the brain data and the WordNet-derived data than the LSA-derived data.

The work reported here builds on earlier work by Suppes and colleagues in which machine learning was employed to recognize brain representations of phonemes, isolated words, sentences, words within sentences, and simple images and their names. That is, the following question was answered affirmatively for EEG and, in one instance, magnetoencephalographic (MEG) data. Can we train a classifier of brain data so that we can predict which word or sentence or word within a sentence the participant is seeing or hearing [6], [7], [8], [9], [10], [11], [12]? Similarly, the following questions were answered. Can we train a classifier on some participants and use it to make predictions for other participants [5], [13]? And, can we train a classifier using words and use that classifier to make predictions for pictures depicting what the words refer to (and vice versa) [14]? A fundamental result that follows from this work is that when brain wave recordings are time-locked to the presentation of words in a sentential context, transformations of segments of those brain waves can be identified as representations (or brain images) of those words. Furthermore, these representations have approximate invariance across test participants and across a variety of sentential contexts. These are clearly representations not of the individuals’ idiosyncratic perceptions of the orthography or sound of the words but of something that could be called the meaning of the words or their semantics.

There have been other recent attempts to study semantics and the brain, also using machine-learning approaches. Many have used functional magnetic resonance imaging (fMRI) data collected while participants were presented with words or word-image pairs. The semantics of a word was represented by its distributional properties in the data set contributed by Google Inc., which consists of English word n-grams and their frequencies in an approximately 1-trillion-word set of web pages [15], [16]. Specifically, for nouns referring to physical objects, their semantics was given by their co-occurrence patterns with 25 manually-selected sensory-motor verbs in the corpus. Taking 60 such noun, fMRI data were collected while participants paid attention to consistent sets of properties of the objects. Using either 58 or 59 words in a training set, statistically significant predictions were made for the semantic category (tool or mammal, for example) of the held-back words relative to each other or to a larger set of words outside the 60. This approach established that semantic features of words could be learned from brain data, thereby allowing a word outside the training set to be correctly classified in terms of its semantic features. More recent work by Just and others sought to reduce the 25-feature representation of the 60 words to a small set of semantic features [17]. The reduction was through factor analysis of the fMRI data to identify locations in the brain that give the best classification of fMRI data for the 60 words.

Other corpora and other ways of selecting the co-occurrence features have been investigated to see if they might offer improved recognition of the Mitchell and others fMRI data [15]. Pereira and others, [18], used semantic features obtained from a large text corpus consisting of articles from Wikipedia pertinent to the specific nouns. Latent Dirichlet allocation (LDA, [19]) gave the co-occurrence features, with words being represented as probabilistic distributions over topics and topics as probabilistic distributions over words. LDA is one of several unsupervised methods that, like LSA, find relations hidden or latent in the text. Kelly and others used parsed data to identify three-part rather than two-part associations, such as *flute-produces-sound,* rather than the simple *flute-sound* [20]. They also included three-part associations involving widely accepted computational and linguistic relations such as *is-a* and *part-of*, as in *Paris is a capital* and *Paris is part of France*. Wikipedia and the British National Corpus provided the language data. In a companion paper, Devereux and others, [21], showed that their semantic representation produces comparable predictions for the fMRI activity of the Mitchell and others dataset [15].

In the work of Murphy and others we see semantics and the brain investigated primarily with EEG data [22], [23], [24]. Using the words in the Mitchell and others experiments as a starting point, EEG data were collected from the presentation of 60 images corresponding to the objects named by the 60 words [15]. These EEG samples, both single-trial and averaged, were classified into two categories (tools and mammals) using co-occurrence data collected for the words describing the images. Several corpora were tested, including a large corpus of newspaper texts that included linguistic information such as part-of-speech tagging and lemmatization. Patterns of co-occurrence were gleaned using singular value decomposition applied to over 22,000 frequent nouns and 5,000 frequent verbs to find the best 25 co-occurrence candidates in place of the pre-selected 25 verbs [15]. Their work also advanced understanding of the time latencies and intervals, frequency bands and scalp locations that improve classification rates, and of the relative advantage of MEG over EEG data. Murphey and others systematically investigated several different distributional models of semantics that could be applied to arbitrary corpora [25]. Of particular interest was the linguistic preprocessing that gave the best results, with dependency parsing and relative position (which word came first in a co-occurrence) proving most effective.

The work of Baroni and others represents efforts to build corpus-based models of semantics that capture something of the nature of the similarity between two words, not just a quantification of that similarity [26]. Properties that could be typed − dogs have tails as *parts*, barking as *behavior*, for example − were of interest and surface grammatical patterns were relied on to pinpoint the potentially interesting co-occurrence patterns. The corpus was a large part-of-speech tagged set of web pages. Outside knowledge sources such as the information in a corpus of child-directed speech and the hypernymy structure of WordNet were used to further constrain co-occurrence candidates.

In Jelodor and others, [27] we find WordNet used as a supplementary source of semantic information for the analysis of the dataset [15]. Several WordNet similarity measures, individually and in combination, were used to compute the similarity of each of the 60 nouns with each of the 25 sensory-motor verbs of Mitchell and others [15]. This approach was handicapped by the fact that in WordNet some similarity measures do not apply to verbs, and some verb-noun comparisons were approximated using noun-noun comparisons, which made sense only when the noun and the verb had strongly consonant meanings. There was also no attempt to limit the WordNet senses to those that were relevant to the specific nouns and verbs, with the highest scoring synset-to-synset similarity score simply being selected. In the end, the highest classification scores obtained in this work were with a combination of the WordNet similarity scores of the 60 nouns with the 25 sensory-motor words and the co-ocurrence features used originally [15].

A move away from corpora is seen in the work of Chan and others [28]. EEG and MEG data were collected during a large number of trials (>1,000) involving a visual and auditory language task in which participants were required to assess the relative size of each of over 500 objects, which were equally divided between living and non-living. The challenge was to learn a binary semantic feature (living or non-living) from the brain data and then correctly classify words in terms of this feature. The amplitude at six time points, using all channels, provided the brain data. The high temporal resolution of EEG and MEG permitted to identify spatial and temporal features of the brain data that improved classification rates and additionally allowed the classification of individual words [28].

Sudre and others also took a corpus-free approach to learning semantic features from brain data [29]. In their study of concrete nouns, scaled values (0 to 5) for semantic features associated with the objects and their names were obtained from a large number of informants. Simultaneous EEG and MEG data were separately collected from participants who were asked Yes/No questions about 20 of these features for 60 objects and their names. Features related to size, manipulability and animacy of the objects proved most effective in predicting MEG data, and these were associated with different spatial and temporal features of the brain data.

In Huth and others we find the WordNet graph structure used to map a large set of categories (1,705), reduced by principal component analysis, onto fMRI data collected while subjects watched videos featuring objects and actions in those categories [30]. The surprising result in this work was how the brain activity related to category responses was so widespread, with activity for some categories localized and for others distributed.

The studies of language and the brain discussed above can each be characterized by whether or not they relied on corpora, specifically the distributional properties of words in corpora, to provide semantic features of the words. For the corpus-based studies, differences exist in the choice of corpus and the strategies used to glean distributional properties, including whether or not linguistic information such as part of speech or WordNet measures were exploited. Those that did not rely on a corpus solicited features either directly or indirectly from language users, relying on a large set of categories and binary or graded membership of categories to specify the semantic model.

Our work draws a comparison between the use of corpus-based distributional properties of words, specifically those provided by LSA, and the semantic information encoded in WordNet. Our interest is not in which gives better predictions of the brain data. It is in the ability of each semantic model – WordNet or the corpus-based distributional semantics of LSA – to represent the network of relations between a given group of words that a participant is considering − {*London, Moscow, Paris, north, south, east, west, Germany, Poland, Russia*}, for example. LSA and other corpus-based distributional semantic models detect relations between words that are latent or hidden in text. WordNet makes these relations explicit based on the judgment of language experts. Ultimately, our interest is in evaluating the extent to which the network of relations we detect in the neural data corresponds to the network of relations represented by LSA compared to WordNet.

The approach taken in this paper is briefly as follows. We use machine learning to predict which word a given brain-data sample is associated with. That is, we classify the brain-data samples into classes that correspond to the words. We then use those classifications to compile confusion matrices, to capture not only which brain-data samples were classified correctly but also, for those that were not correctly classified, the words they were incorrectly associated with. This approach derives from the well-known experiments in Miller and Nicely in which confusion matrices were used to deduce important information about the perception of phonemes [31]. In our analysis, the pattern of mistaken predictions for the brain data is used to tell us something important about the perception of words in the brain. We can then compare what we learn about these perceptions with the two semantic models, WordNet and LSA.

The main contribution of this paper is in the new method it introduces by which structural similarities between brain data and linguistic data can be compared at the semantic level. Included in this method is a way to measure the strength of the structural similarities found between the brain data and the linguistic data, which allows us to assess the relatively better fit of the brain data with one semantic model over another.

## Materials and Methods

### 2.1 Brain Data

Brain data comes from the experiments described in Suppes and others [5], [14]. In the experiment of interest, 48 spoken sentences about the geography of Europe were presented to nine participants in 10 randomized blocks, with all 48 sentences occurring once in each block. The 48 sentences were statements about commonly known geographic facts of Europe, with half of the sentences true, half false, half positive, and half negative (e.g., *The capital of Italy is Paris* and *Paris is not east of Berlin*). The possible forms of these trigger sentences are: *X is [not]W of Y*, *W of Y is [not]X, X is [not]Z of X, Y is [not] Z of Y*, where *X *<$>\raster="rg1"<$> {*Berlin, London, Moscow, Paris, Rome, Warsaw, Madrid, Vienna, Athens*), *Y* <$>\raster="rg1"<$> {*France, Germany, Italy, Poland, Russia, Austria, Greece, Spain*), *W* <$>\raster="rg1"<$> {*the capital, the largest city*}, *Z* <$>\raster="rg1"<$> {*north, south, east, west*}, and *[not]* indicates the optional presence of *not*. The maximum sentence length was 2.59 s and the minimum 1.35 s, with the interval from sentence onset to sentence onset 4.05 s. In a related experiment using the same sentences, the words were visually displayed one by one on a computer screen. The same timing was used in the auditory presentation of the sentences, providing a start and end time for each word. The sampling rate was 1,000 Hz and the data were down-sampled 16 times, giving an effective sample rate of 62.5Hz.

With our focus on individual words in their sentential contexts, we extracted segments of the brain-wave data for each word of interest from every trial in which that word appeared. For classifying the wave-form segments, we needed to produce data samples of equal length. We let each word’s data sample begin approximately 160 ms after the auditory onset of the word, that is, 10 data points beyond the word’s initial start point. To determine the end point, we extended each word’s data sample by 50 data points (that is, approximately 800 ms) and calculated the average length of the resulting data samples. This average length then determined the end point of each word’s data sample. The data samples from all fifteen channels were concatenated, producing a single brain-data sample for each occurrence of a word. The choice of start and end points was informed by work showing the N400 component of the event related potential (ERP) to be associated with semantic processing and integration [32], [33], [34]. Furthermore, recent work by Brennan and Pylkkänen confirmed the long-held assumption that since sentences appear to be understood incrementally, word-by-word, brain activity time-locked to the presentation of words in sentences can be analyzed to tell us something about language processing [35]. They additionally found that the activity 250 ms after word onset can be associated with the processing of words in sentences as opposed to words in lists.

The 21 country names, city names, and the four directions or relative locations are the words of interest for our analysis. We selected three city names, three country names and the four relative location words for classification as a group. The linguistic data for the ten words {*London, Moscow, Paris, north of, south of, east of, west of, Germany, Poland, Russia*} and the brain data for one subject (S18) are used to illustrate the methods throughout the paper. We chose S18 because that participant gave good classification rates. We use the data from this subject and the one set of ten words consistently throughout the paper to eliminate any possibility of cherry-picking participant-word samples in each instance to support our points. Results for all nine participants and all 21 words are reported in the paper, however, and form the basis for our conclusions. The words occurred within the trials with the frequencies here shown in parentheses: *Berlin* (70), *London* (80), *Moscow* (90), *Paris* (80), *Rome* (80), *Warsaw* (80), *Madrid (70),* Vienna (40), *Athens* (50), *France* (40), *Germany* (40), *Italy* (40), *Poland* (40), *Russia* (50), *Austria* (40), *Greece* (40), *Spain* (50), *north* (60), *south* (70), *east* (60), *and west* (70). Material S1 gives the trigger sentences and the timing of the presentation of each word for each sentence.

### 2.2 Models and Methods of Analysis

#### 2.2.2 Structural models

The first step in constructing structural models for the brain data is to analyze the data using a statistical model that predicts to which class a brain-data sample belongs, where each class corresponds to one of the 21 word types. In order to classify the segments of data obtained from the individual trials, we use a linear discriminant model in a 5-fold cross-validation loop. The dimensionality of the data was reduced using a nested principal component analysis. Ocular artifacts were removed from the brain-data samples using blind source separation. The procedure is described in detail by Perreau-Guimaraes and others [11]. This classifier resulted from 10 years experimentation with different kinds of classifiers. It gave consistently higher recognition rates and it was used to derive the results that led to the current work [12].

For our 10 sample words, each of 640 brain-data samples is classified as a representation of one of the 10 words. More generally, the T brain-data samples s_1_, s_2_, …, s_T_ are classified into the N classes ω_1_, ω_2_, …, ω_N_. In this process, we do not directly compare the samples to each other to judge their similarity; rather we use a set of N prototypes, and these are what are designated by ω_1_, ω_2_, …, ω_N_. If test sample s_i_ is classified as ω_i_ then s_i_ and ω_i_ have a minimal similarity difference compared to the other possible classifications.

**Figure 1:**
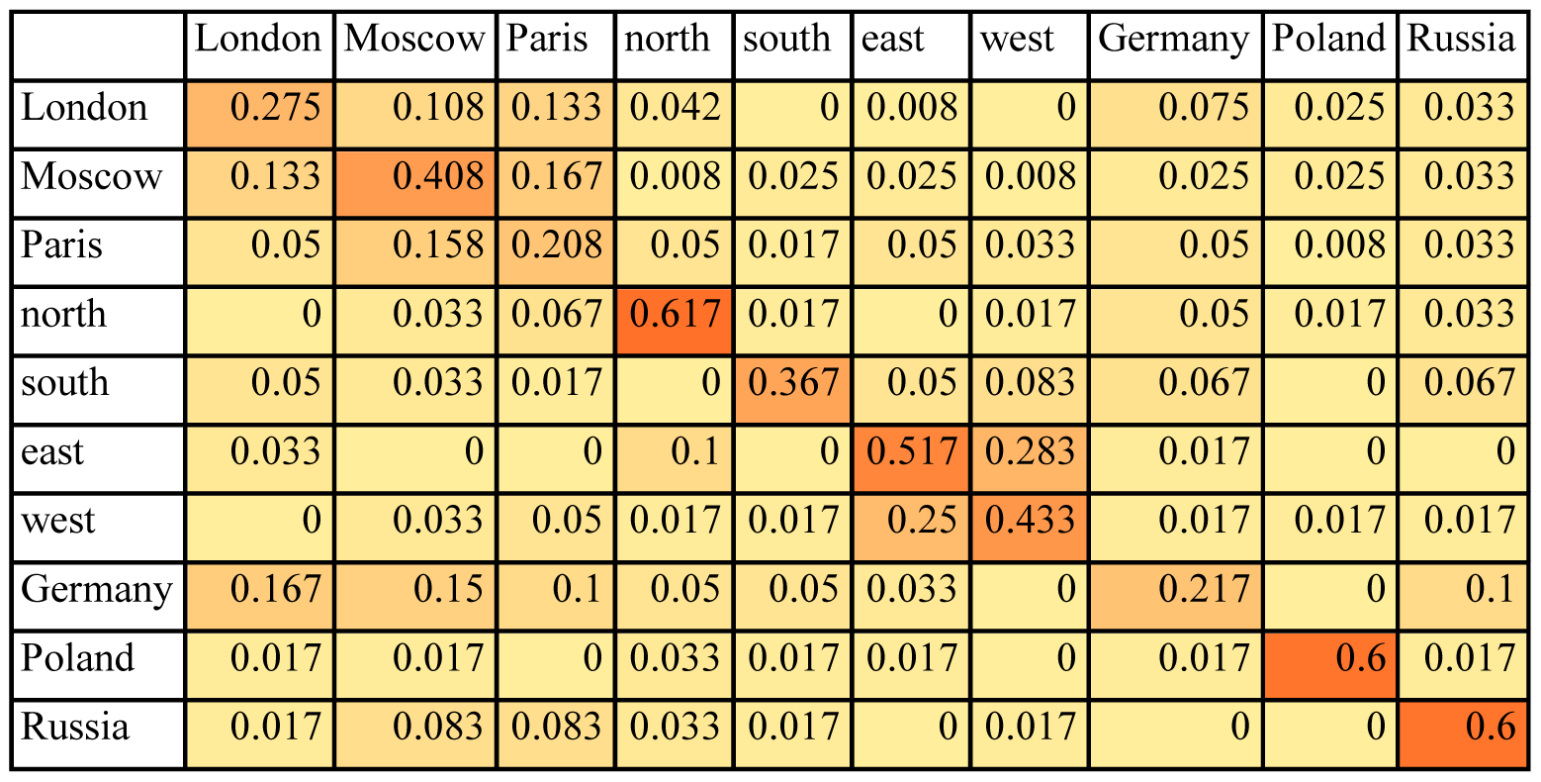
Conditional probability density estimates (shown as a heat map) computed from the confusion matrix resulting from the classification of 640 brain wave samples from S18 for the set of words {*London, Moscow, Paris, north, south, east, west, Germany, Poland, Russia*}. This table was taken from [12].

Let M  =  (m_ij_) be the confusion matrix for a given classification task, where m_ij_ is the number of test samples from class ω_i_ classified as belonging to class ω_j_. By computing the relative frequencies m_ij/_ Σ_j_m_ij_ we obtain N-by-N estimates for the conditional probability densities that a randomly chosen test sample from class ω_i_ will be classified as belonging to class ω_j_. Figure 1 gives the conditional probability estimates computed from the confusion matrix resulting from a single-trial classification of the brain data for {*London, Moscow, Paris, north, south, east, west, Germany, Poland, Russia*} from participant S18. Following the conventions for heat maps, the higher the value of each element in the matrix the darker its shading.

Let these conditional probability estimates be given by the matrix P = (p_ij_). For each class ω_i_ we then define a quaternary relation R such that ω_i_ ω_j_ R ω_i_ ω_k_ if and only if p_ij_ <p_ik_, that is, if and only if the probability that a randomly chosen sample from class ω_j_ will be classified as belonging to class ω_i_ is smaller than the probability that a randomly chosen test sample from class ω_k_ will be classified as belonging to class ω_i_. R is an ordinal relation of similarity differences, a partial order that is irreflexive, asymmetric, and transitive. It can of course be simplified to a ternary relation R* (ω_i_ ω_j_ ω_k_). For simplicity we continue to use R for this ternary relation.

Along the lines of the approach for the axiomatization of the theory of differences in utility preference or the theory of differences in psychological intensity, we put forward the relational structure (A, R), which is constructed from R and the finite set A of classes ω_i_ together with the real-valued functions given by the inequalities p_ij_ <p_ik_ [36]. (Specifically, the function defined by these inequalities is the function f defined on A such that xy R uv iff f(x) − f (y )<f (u) − f (v ), with f(x) − f(y) represented by p_xy_.) Note that an ordinal relation of similarity differences, a partial order that is irreflexive, asymmetric, and transitive, is defined for each class ω_i_. We therefore have N partial orders and the notation (A, R) will from here on be understood to refer to a structure with N partial orders defined on the set A.

At this point we have a formal characterization of the brain data from the experiment. What of the linguistic data? The focus was on the recognition of phonemes and words [12]. Linguistic data were provided by the well-known Miller and Nicely experiment in which participants heard 16 English consonants under various conditions of noise or other distortion and then reported their perceptual judgments by classifying the consonants [31]. The confusion matrix of phonemes arising out of these perceptual judgments gives rise to a relational structure (A’, R’) constructed from R’ (more precisely from N such relations) and the finite set A’ of classes ω’_i_ together with the real-valued function (more precisely, the N such functions) given by the inequalities p’_ij_ <p’_ik_. The two relational structures (A, R) and (A’, R’) provide the means by which comparison can be made between the brain data and the linguistic data. For the Miller and Nicely experiments, the linguistic data were provided by human judgments of similarity. For the geography sentence experiments, the linguistic data are provided by similarity measures derived from two models of semantic similarity, as we describe in Section 2.3.

#### 2.2.3 Comparing structural models of brain and semantic data

Isomorphism between (A, R) and (A’, R’) would constitute the strongest measure of similarity, but isomorphism is almost certainly too strong a requirement for brain data obtained under current experimental conditions. The confusion matrices of the brain and linguistic data could be compared directly using a statistical test such as the chi square, for example. And cluster or similarity trees can be computed from these matrices, allowing visual assessment of the congruence between the brain and linguistic data. However, direct comparison of the confusion matrices, whether visually or using a single measure of quantitative fit, is both too strong under current experimental conditions and too weak. It is too strong because data derived from electrophysiological measurements on the scalp almost certainly present an incomplete picture of brain activity. Direct visual or single-measure comparison is too weak because it tells little of the precise nature and strength of any congruence there might be between the brain and linguistic data. Instead we have devised a procedure that compares the partial orders of the two relational structures described in the previous section. This approach, we submit, has a stronger theoretical basis and offers a more detailed view of the similarities that do hold between brain data and perceptual or linguistic data.

We derive from the NxN matrix of conditional probability densities for the brain data N strict partial orders R^B^ _i_ (i = 1,…,N) of the similarity differences of the ordered pairs, one ordering for each of the N classes into which the samples are classified. Similarly for the perceptual data related to phonemes or, the focus of this paper, the semantic data related to the words. We derive from the perceptual or semantics matrix N strict partial orderings R^P^ _i_ (i = 1,…,N) of the similarity differences of the ordered pairs, one ordering for each of the N classes into which the samples are classified. For simplicity, we omit the index i in the description that follows. R^B^ and R^P^ are understood to refer to the orderings for any one of the N classes.

The question now is how to compare the two partial orders. For each class ω_i_, we first compare R^B^ and R^P^ to see if they are order isomorphic. Order isomorphism is defined in terms of the partially ordered sets (or posets) corresponding to the partial ordering relations. Two partially ordered sets X and Y are order isomorphic iff there is a one-to-one and onto mapping f from X to Y such that for all x_1_, x_2_ ∈ X, the ordered pair (x_1_, x_2_) ∈ X iff the ordered pair (f(x_1_), f (x_2_)) ∈ Y. That is, the mapping f between the two posets is bijective and both f and f ^−1^ are order preserving. If f also preserves the mapping between the classes in the relational structure for the brain data and those in the relational structure for the linguistic data, order isomorphism establishes a strong notion of similarity [37].

Order isomorphism at the level of the individual classes is more likely under current experimental conditions than isomorphism between the two full relational structures. Nonetheless, what is required to compare the brain and linguistic data is a generalization of the notion of order isomorphism to the notion of *invariant partial order*. An invariant partial order for the brain and linguistic data is the intersection of the partial order for the brain data, R^B^, and the partial order for the perceptual data, R^P^. Each intersection R^INV^ = R^B^ R^P^, one for each of the classes into which the samples are classified, is also a partial order that is irreflexive, asymmetric, and transitive. We devise a way to judge the strength of the structural similarity represented by these invariant partial orders.

We compute the Spearman rank correlation coefficient of the two partial orders R^B^ and R^P^ that give rise to each invariant partial order. Each invariant partial order for which the contributing R^B^ and R^P^ partial orders have a statistically significant correlation between them is counted as a significant invariant partial order. It is these significant invariant partial orders that constitute the structural similarity between the brain and linguistic data, and the total number of such significant partial orders is a measure of the strength of that structural similarity.

We have restricted ourselves to strict partial orders so far but it can be the case that confusion matrices produced by the brain or perceptual data contain ties in a row, that is, for any class ω_i_. If these equal numerical scores are permitted in the partial order, the result is a strict weak ordering instead of a strict ordering and the property of irreflexivity does not hold. Instead of permitting ties in the data to give rise to weak strict orders, a possibly more realistic response to equal numerical scores in the experimental context lies with semiorders. A semiorder requires that two numerical scores be within some threshold of each other before they are compared. Introduced in mathematical psychology, semiorders are intransitive and used as a model of human preference that permits nontransitive indifference [38], [39]. If the threshold is allowed to vary from one score to another, the order is an interval order. Semiorders or interval orders can prove useful in comparing relational structures for brain and perceptual data, [12]; in this paper, however, Suppes and others restrict ourselves to strict partial orders.

In summary, the procedure by which structural similarity between brain and linguistic data is judged is as follows. The similarity differences from the linguistic data and the brain data respectively are presented in the form of two confusion matrices. For each matrix, conditional probabilities are constructed from which we define N strict partial orders for the brain data and N for the linguistic data. For each N, the intersection of the brain and language partial orders give rise to one or more invariant partial orders with respect to the brain and linguistic data. Those invariant partial orders for which the contributing brain and language partial orders are significantly correlated, as measured by the Spearman rank correlation coefficient, constitute the structural similarity between the brain and perceptual data. The total number of significant invariant partial orders gives a measure of the strength of that structural similarity.

### 2.3 Semantic Data

#### 2.3.1 WordNet-based model of similarity

WordNet is a lexical database of English compiled by experts using lexicographic practices to identify word senses, define relations between those senses, and compose glosses (or definitions) of those senses. In WordNet, nouns, verbs, adjectives and adverbs are each grouped into sets of cognitive synonyms (called synsets), each expressing a distinct concept [1], [2], [40]. The synsets are related to each other, primarily through the hypernymy and hyponymy relations for nouns. Other relations in WordNet are part-whole (holonym), member of (meronym), has instance, and so on. Hyponymy, often referred to as the *is–a* relation in computational discussions, is defined as follows: a concept represented by a lexical item L_i_ is said to be a hyponym of the concept represented by a lexical item L_k_ if native speakers of English accept sentences of the form "An L_i_ is a kind of L_k_." Conversely L_k_ is the hypernym of L_i_. A hypernym is therefore a more general concept and a hyponym a more specific concept. The WordNet database has the structure of a directed acyclic graph, and the node for one concept c_x_ is said to be an ancestor of the node for another concept c_y_ if c_x_ can be reached from c_y_ by traversing one or more relational links, known as paths or edges. In WordNet one root node subsumes all of the noun concepts, and similarly for verbs, adjectives and adverbs.

The well-known example of *canaries can fly*, and others like it, suggests that humans represent words and their semantic relations to each other something like WordNet does. We can quickly verify that *canaries can sing* because a canary is a songbird, with only one level of hyponymy between. But to verify that *canaries can fly*, two levels of hyponymy must be traversed and to verify that *canaries have skin* several levels. These two judgments take correspondingly more time. Words as represented in WordNet have multiple senses, each sense being part of one or more synsets. The WordNet senses for the city names, the country names, and the four direction or location terms (*east, west, north and south*) are listed in Material S2. For each word, the senses relevant to the geography sentences are listed first, with the non-relevant senses shown indented beneath. There are two senses of *London* in WordNet, for example. The first, designated London#1, concerns the capital of Britain, the second, designated London#2, the writer. Only the first sense of the word *London* is relevant to the geography sentences of our experiment.

**Figure 2:**
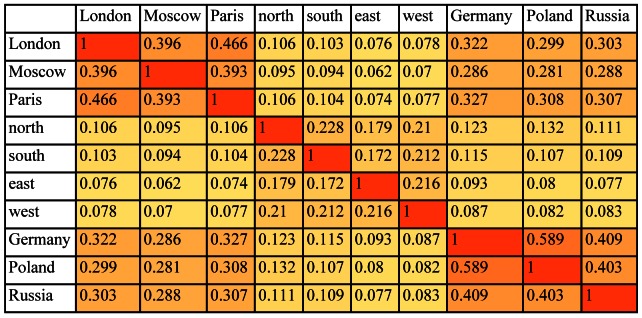
Semantic similarity matrix (shown as a heat map) derived from WordNet for the set of words {*London, Moscow, Paris, north, south, east, west, Germany, Poland, Russia*} using senses relevant to the geography of Europe.

**Figure 3:**
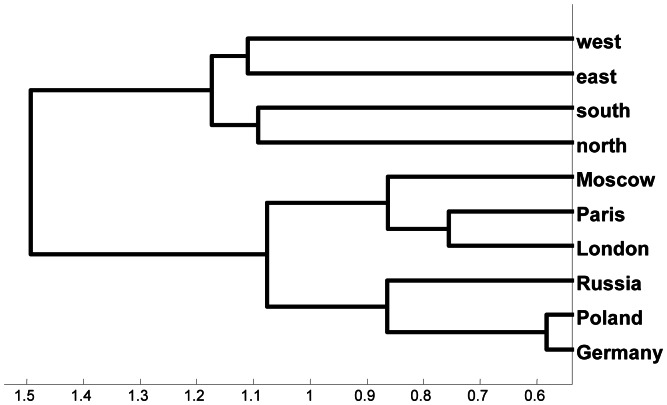
Hierarchical cluster tree computed from the WordNet-based semantic similarity matrix for {*London, Moscow, Paris, north, south, east, west, Germany, Poland, Russia*} given in Figure 2.

Several different WordNet-based measures have been proposed to represent the degree to which two words are related. A detailed discussion of such measures [41] and online access is given to a program that computes these [42], [43]. (Pedersen, Patwardhan, Michelizzi, 2004). Three main approaches have dominated. The first uses path length between two synsets measured along WordNet’s taxonomic (*is-a*) paths, the second uses the notion of information content, a corpus-based frequency-related measure, and the third is a vector-space-based approach that uses the words in the definitions (or "glosses") of the synsets. We combined five measures in our computations of similarity to take account of these different approaches, averaging the results from measures based on path-length, information-content, and vector-space, along with hybrids of these. Each similarity measure captures something important about the relations between words as represented in WordNet. Many studies have compared these and other measures of relatedness in WordNet, assessing their effectiveness for tasks as varied as word-sense disambiguation, information retrieval, indexing, and spelling correction, as done in Budanitsky and Hirst [41]. However, none emerges as clearly superior as a general measure of similarity-oriented relatedness. Recent work by Ballatore and others, furthermore, specifically compared the performance of several combinations of WordNet similarity scores against individual scores and found a clear pattern of success (that is, fidelity to human judgments) for combined scores [44]. Coincidentally, their work looked specifically at geography-related word. Further details of the five individual measures we used are given in Material S3. In Figure 2 we show the results for all 10 words averaged over all relevant senses. Figure 3 gives a hierarchical cluster tree computed for the data in Figure 2 using furthest (also known as complete) distance. It shows the expected groupings of the city names, country names, and location or direction names.

#### 2.3.2 Statistical method for extracting latent semantic relations

**Figure 4:**
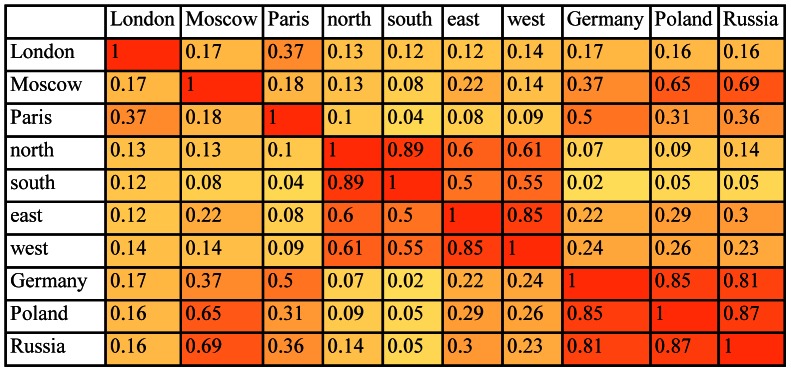
Similarity scores computed using LSA for the words {*London, Moscow, Paris, north, south, east, west, Germany, Poland, Russia*} shown as a heat map.

**Figure 5:**
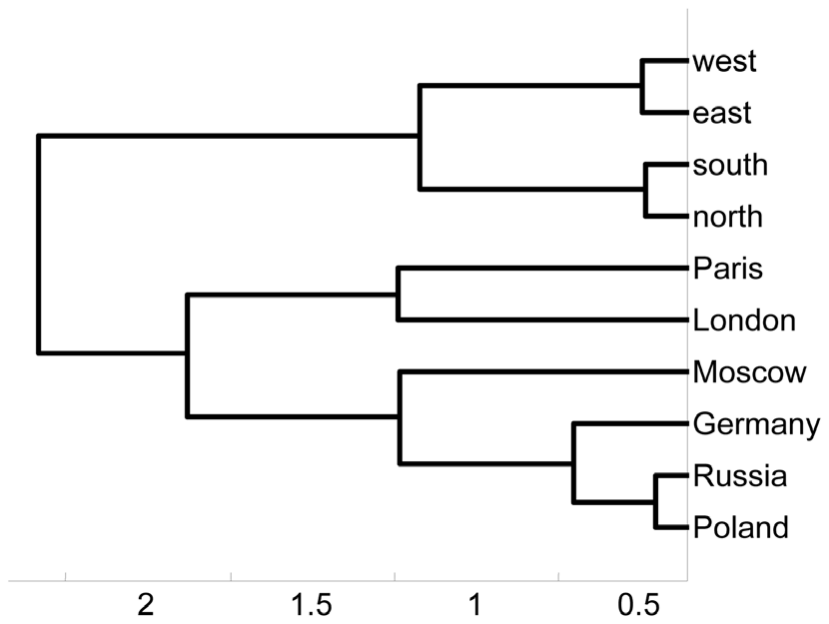
Hierarchical cluster tree computed from the LSA scores of similarity for {*London, Moscow, Paris, north, south, east, west, Germany, Poland, Russia*} given in Figure 4.

LSA is a statistical technique for extracting from large collections of documents a measure of how similar two words are to each other in terms of patterns of their co-occurrences within those documents [4], [3], [45]. The underlying idea is that if you take into account all the contexts in which a word does and does not appear within a set of documents, you capture something important about that word’s meaning in those documents. LSA has a plausible interpretation as a psychological theory of similarity and knowledge. The similarity judgments produced by latent semantic analysis have been shown to correspond to some extent to human judgments of similarity. For example, after training on about 2,000 pages of English text, it scored as well as average test-takers on the synonym portion of the Test of English as a Foreign Language (TOEFL). LSA’s judgments of similarity are derived from the specific document set used in the computations, a restriction that can be of practical advantage. For example, after training on a psychology textbook it achieved a passing score on a multiple-choice exam. LSA as generally implemented does not use syntactic information, which most likely limits what it can capture about semantic similarity, but there is no reason such information cannot be incorporated into an LSA-type model. We used the application at http://lsa.colorado.edu/(accessed January 15, 2013) to compute similarity matrices in term space for our set of words. See Figure 4. The computation was based on texts of general reading up to 1st year college level and a maximum of 300 factors was permitted in the analysis. Figure 5 gives the similarity tree computed from Figure 4. It shows the expected groupings of the city names, country names, and direction terms except for a displacement of *Moscow* into the country names.

### 2.4 Structural Models of Similarity between Brain Data and Semantic Data

We now have two structural representations, one of the brain data and the other of the semantic data. We compare them.

#### 2.4.1 Invariant partial orders

**Figure 6:**
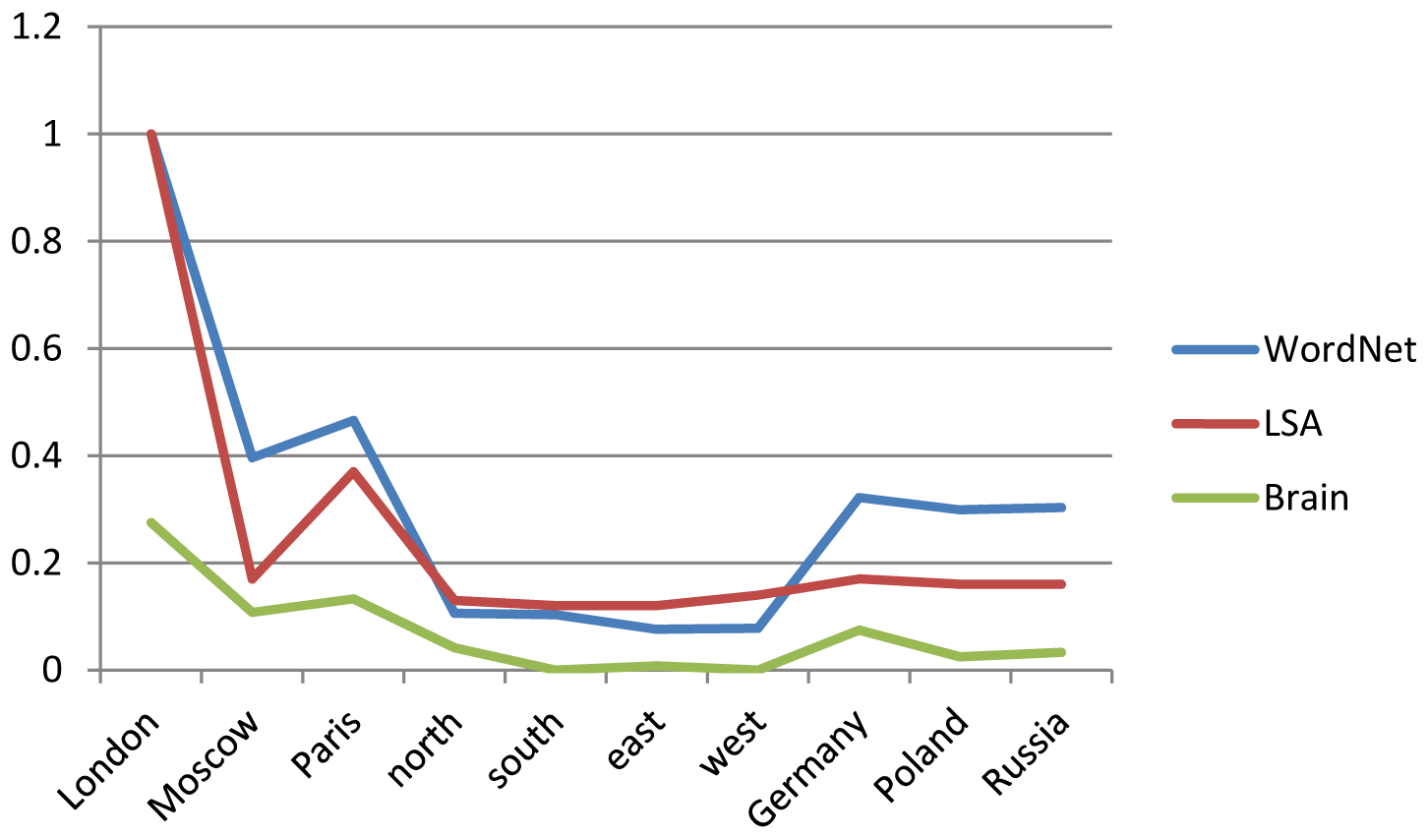
WordNet-based and LSA-based semantic similarities and EEG conditional probability estimates for *London* relative to *London*, *Moscow*, *Paris*, *north*, *south*, *east*, *west*, *Germany*, *Poland*, and *Russia*. Data taken from Figure 1, Figure 2, and Figure 4.

**Figure 7:**
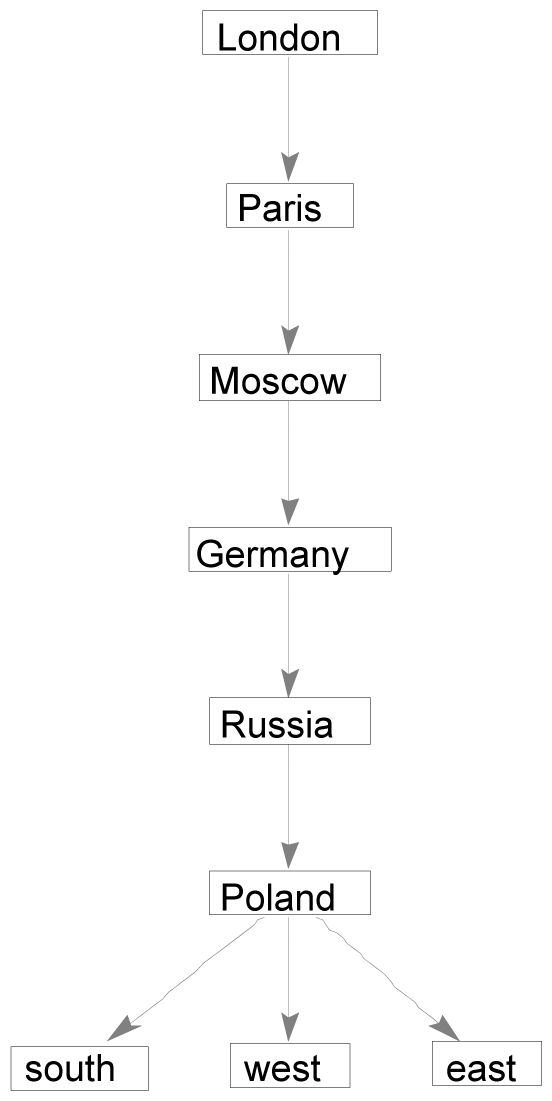
Joint invariant partial order for *London* relative to the set of words {*London, Moscow, Paris, north, south, east, west, Germany, Poland, Russia*}. Brain data derived from FIGURE 1 and semantic data from the WordNet data in Figure 2.

We start with the conditional probability density estimates in Figure 1 derived from the brain data and the similarity scores derived from WordNet (Figure 2) and LSA (Figure 4). Line graphs of the conditional probability densities and the semantic similarities for each word (that is, each row of the two tables) give a sense of how the linguistic data and the brain data appear to track each other. See Figure 6 for the example of *London*. (Data for the other words can be found in Figure S1 a–j.) The Spearman rank correlation for the brain data and the WordNet-derived data in Figure 6 is 0.99, with one-sided significance of 1.84E-10. The Spearman rank correlation for the brain data and the LSA-derived data in Figure 6 is 0.8758 with one-sided significance of 8.9494e-004. The Spearman rank correlation, as an indication of the strength of the relationship between two partial orders, is a statistic of interest. But what is of primary interest based on the approach described in section 2.4 are the ordinal relations of similarity differences, partial orders that are irreflexive, asymmetric, and transitive, that are *invariant* with respect to the semantic and brain data. For *London*, the WordNet partial order is given by the 10-element sequence {*London Paris Moscow Germany Russia Poland north south west east*}. The brain data yields two nine-element sequences {*London Paris Moscow Germany north Russia Poland east south* } and {*London Paris Moscow Germany north Russia Poland east west*} arising from the tie between the scores for *south* and *west*. See Table 1. The partial orders that are invariant with respect to the brain data and WordNet-derived data are the three seven-element sequences {*London Paris Moscow Germany Russia Poland south*}, {*London Paris Moscow Germany Russia Poland west* } and {*London Paris Moscow Germany Russia Poland east*}. These three invariant partial orders jointly constitute the similarity between the brain and semantic data for *London* relative to the set of words {*London, Moscow, Paris, north, south, east, west, Germany, Poland, Russia*}. A graphical representation is given in Figure 7.

**Table 1:** Partial orders for *London* derived from the WordNet semantic similarities of Figure 2 and the conditional probability estimates for the brain data of Figure 1.

London				
Language data		Brain data		
London	1.000	London	0.275	
Paris	0.466	Paris	0.133	
Moscow	0.396	Moscow	0.108	
Germany	0.322	Germany	0.075	
Russia	0.303	north	0.042	
Poland	0.299	Russia	0.033	
north	0.106	Poland	0.025	
south	0.103	east	0.008	
west	0.078	south	0.00	
east	0.076	west	0.000	

**Figure 8:**
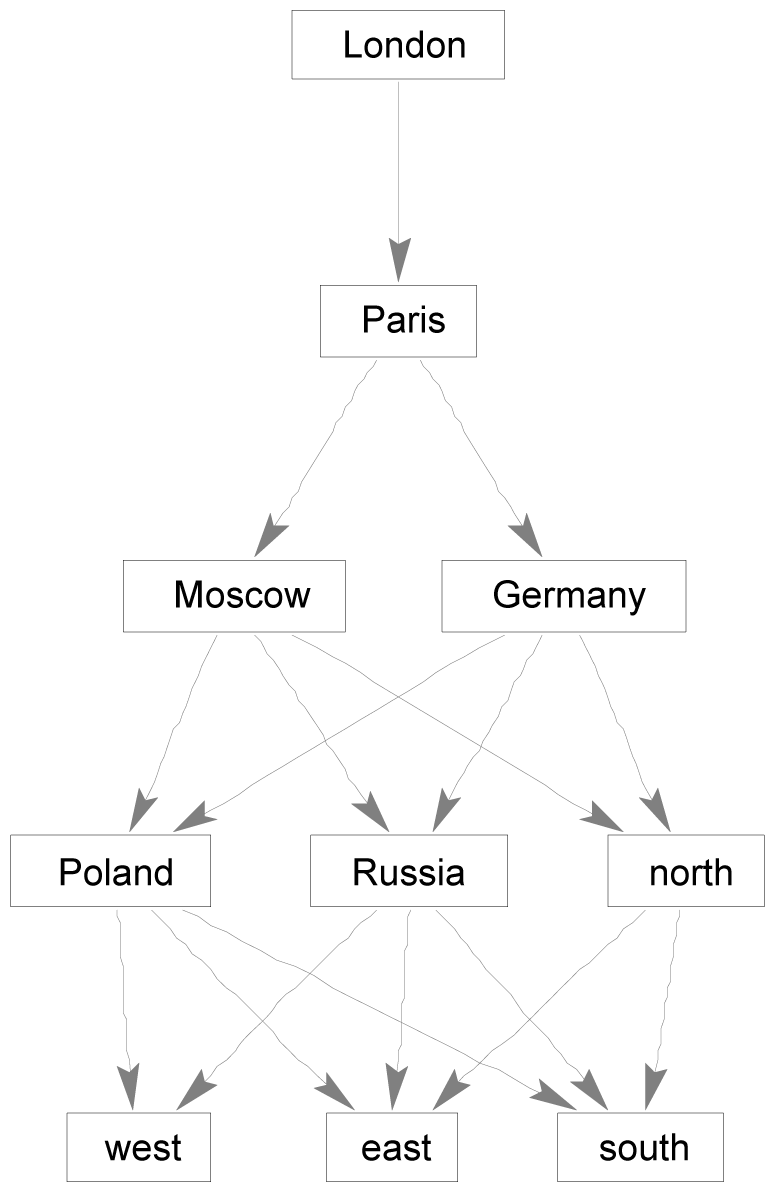
Joint invariant partial order for *London* relative to the set of words {*London, Moscow, Paris, north, south, east, west, Germany, Poland, Russia*}. Brain data derived from Figure 1 and the LSA data from Figure 4.

Looking now at the partial orders derived from the LSA linguistic data and, again using *London* as an example, we find the partial orders that are invariant with respect to the brain data and these linguistic data. The LSA data yield eight seven-element sequences. The brain data yield the same two nine-element sequences as before. There are 16 partial orders that are invariant with respect to the brain and LSA-derived data. They jointly constitute the similarity between the brain and LSA semantic data for *London* relative to the set of words {*London, Moscow, Paris, north, south, east, west, Germany, Poland, Russia*}. A representation of the graphical join of these invariant partial orders is given in Figure 8.

### 2.5 Computing Invariant Partial Orders

**Figure 9:**
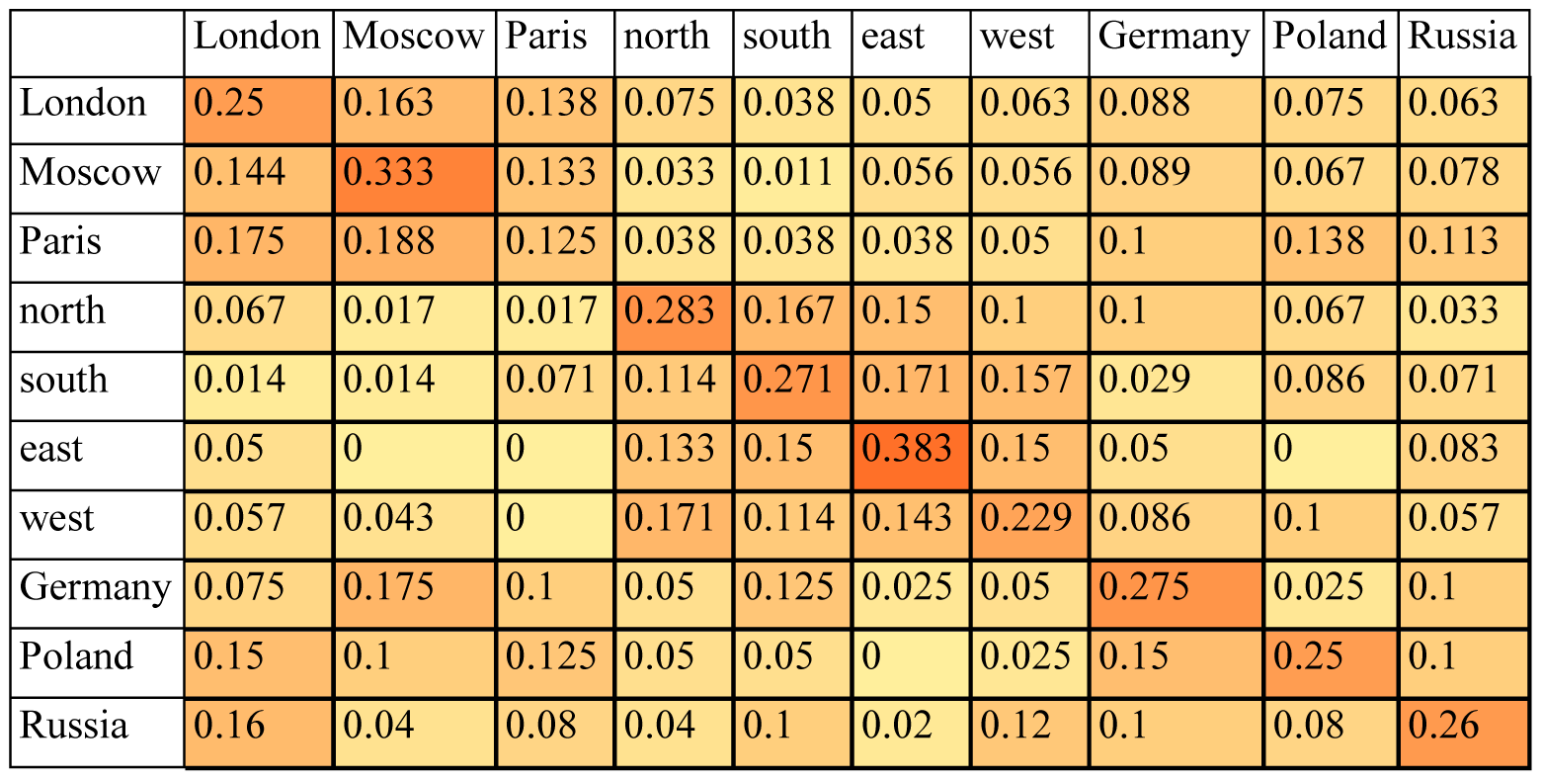
Conditional probability density estimates (shown as a heat map) from a new single-trial classification of the brain data (S18) for {*London, Moscow, Paris, north, south, east, west, Germany, Poland, Russia*}.

In this section we turn to the task of computing the invariant partial orders for the brain and linguistic data. To illustrate we use the semantic similarity matrix of Figure 2– that is, the Wordnet-derived semantic data for {*London, Moscow, Paris, north, south, east, west, Germany, Poland, Russia*} – and the conditional probability density estimates presented in Figure 9 below. The estimates in Figure 9 were computed using, as before, the linear discriminant model of Perreau-Guimaraes and others to classify the 640 single-trial data samples for the 10 geography words for participant S18 [11]. All computations described here were performed using the MATLAB programming environment. We use this new classification of the same ten words for the same participant to illustrate an important computational point that is also an important point about the brain data. We return to this point in section 2.6.

As stated earlier, for each word ω we define a ternary relation R’ such that R’(ω, ω_1_, ω_2_ ) if and only if with respect to word ω the conditional probability for word ω_1_ is smaller than the conditional probability for word ω_2_, that is if and only if ω_1_’s similarity difference with ω is smaller than ω_2_’s similarity difference with ω, or in terms of semantic similarities ω_1_ is more similar to ω than is ω_2_. R’ is an ordinal relation of similarity differences, a partial order that is irreflexive, asymmetric, and transitive.

**Figure 10:**
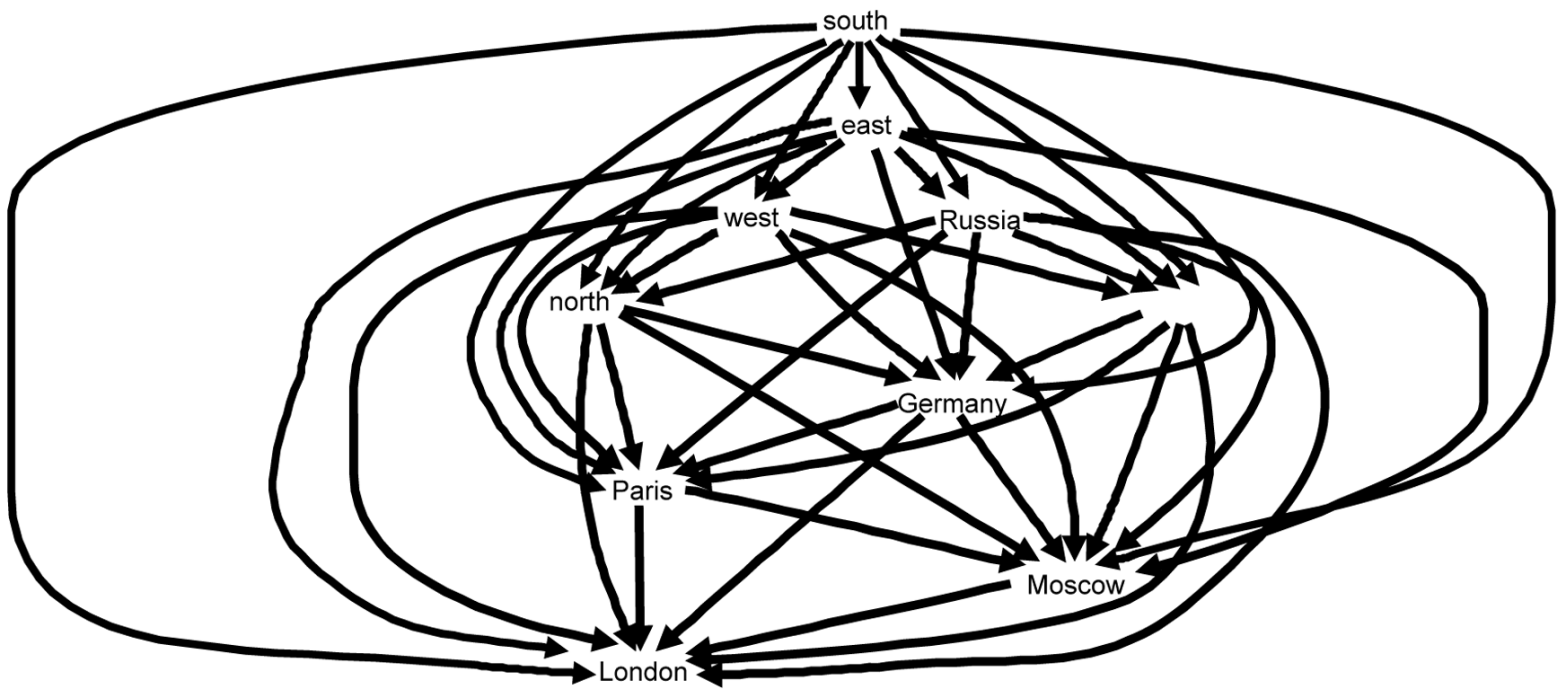
Ordinal relation of similarity differences represented as a directed acyclic graph CG^B^_London_ for *London* relative to the set of words {*London, Moscow, Paris, north, south, east, west, Germany, Poland, Russia*}, derived from the probability density estimates for the brain data in Figure 1.

**Figure 11:**
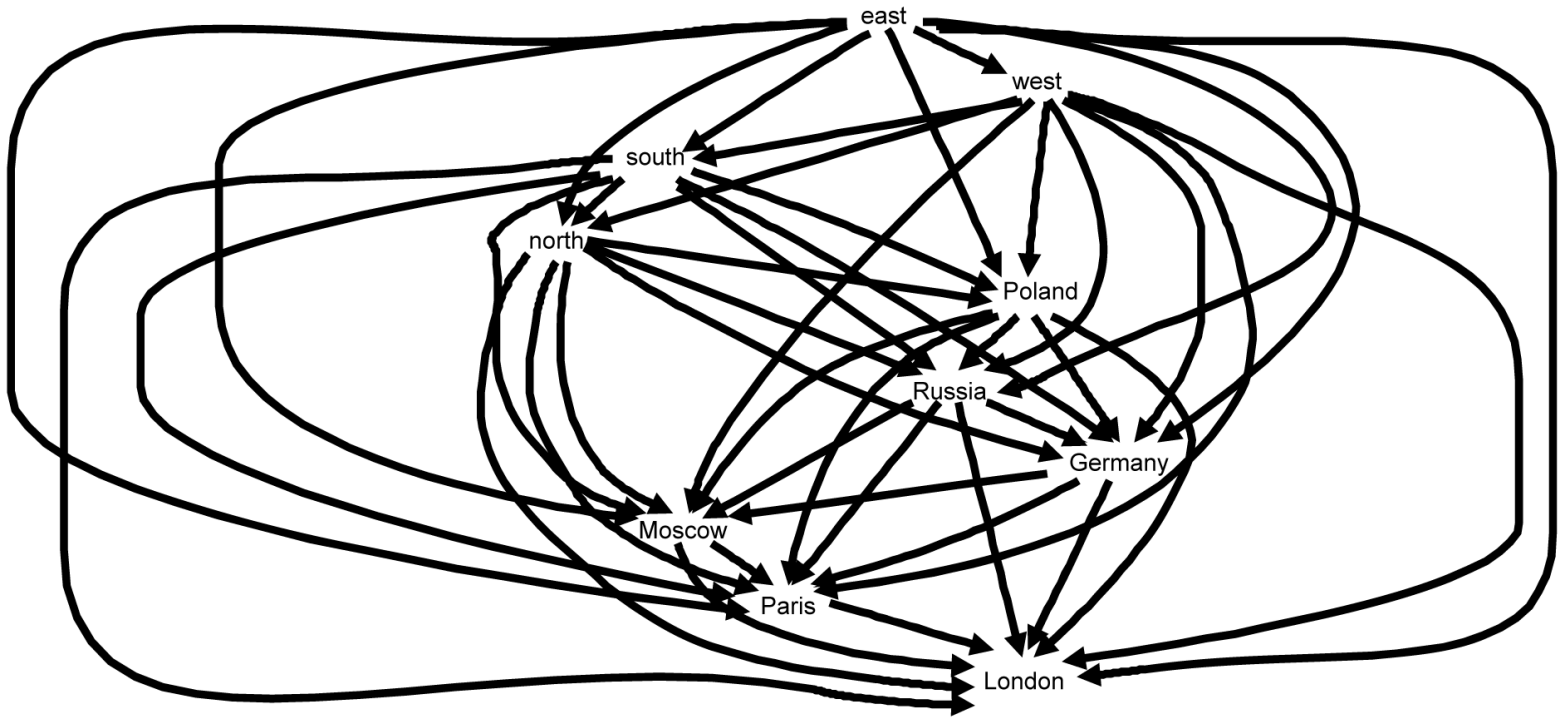
Ordinal relation of similarity differences represented as a directed acyclic graph CG^S^_London_ for *London* relative to the set of words {*London, Moscow, Paris, north, south, east, west, Germany, Poland, Russia*}, derived from the WordNet data in Figure 2.

R’ can be represented computationally using a connection matrix C_ω_ where C_ω_(ω_1_, ω_2_) = 1 if and only if with respect to word ω the conditional probability for word ω_1_ is smaller than the conditional probability for word ω_2_, that is if ω_1_’s similarity difference with ω is smaller than ω_2_’s similarity difference with ω. Similarly for the semantic similarity matrix of Figure 2. These connection matrices can be computed automatically in a straightforward manner from Figure 2 and Figure 9. The connection matrix C^B^_London_ for *London* for the brain data is given in Table 2. The connection matrix C^S^_London_ for *London* for the semantic data is given in Table 3. Highlighted entries show where the partial orders differ. A directed acyclic graph can be derived from the connection matrix for a word ω, with an arrow going from node ω_1_ to node ω_2_ if and only if ω_1_’s similarity difference with ω is smaller than ω_2_’s similarity difference with ω. Figure 10 shows the connection graph CG^B^_London_ for the brain data for *London*, and Figure 11 the connection graph CG^S^_London_ for the semantic data. These connection graphs may appear too complex to be potential models of semantic relations as they are represented in the brain. However, although these structures are visually complex in graph form, the connection matrices to which they are logically equivalent are structurally simple and amenable to simple computations. More will be said in the Discussion about the role such networks may play in accounts of how the brain processes language.

**Table 2:** Ordinal relation of similarity differences represented as a connection matrix C^B^_London_ for *London* relative to the set of words {*London, Moscow, Paris, north, south, east, west, Germany, Poland, Russia*} shown by initial letters, derived from the probability density estimates for the brain data in Figure 9.

	L	M	P	n	s	e	w	Ge	Po	Ru
L	0	0	0	0	0	0	0	0	0	0
M	1	0	**0**	0	0	0	0	0	0	0
P	1	**1**	0	0	0	0	0	0	0	0
n	1	1	1	0	0	0	0	1	**0**	**0**
s	1	1	1	1	0	**1**	**1**	1	1	1
e	1	1	1	1	**0**	0	1	1	1	1
w	1	1	1	1	**0**	0	0	1	1	**0**
Ge	1	1	1	0	0	0	0	0	0	0
Po	1	1	1	0	0	0	0	1	0	**0**
Ru	1	1	1	**1**	0	0	0	1	**1**	0

**Table 3:** Ordinal relation of similarity differences represented as a connection matrix C^S^_London_ for *London* relative to the set of words {*London, Moscow, Paris, north, south, east, west, Germany, Poland, Russia*} shown by initial letters, derived from the WordNet data in Figure 2.

	L	M	P	n	s	e	w	Ge	Po	Ru
L	0	0	0	0	0	0	0	0	0	0
M	1	0	**1**	0	0	0	0	0	0	0
P	1	**0**	0	0	0	0	0	0	0	0
n	1	1	1	0	0	0	0	1	**1**	**1**
s	1	1	1	1	0	**0**	**0**	1	1	1
e	1	1	1	1	**1**	0	1	1	1	1
w	1	1	1	1	**1**	0	0	1	1	**1**
Ge	1	1	1	0	0	0	0	0	0	0
Po	1	1	1	0	0	0	0	1	0	**1**
Ru	1	1	1	**0**	0	0	0	1	**0**	0

To return to our task of computing the structural similarity between brain and linguistic data, connection graphs allow isomorphisms to be computed automatically using standard functions in the MATLAB programming environment. In MATLAB, biograph objects CG^B^_London_ and CG^S^_London_ are constructed from connection matrices C^B^_London_ and C^S^_London_ using the statements CG^B^_London_ = *biograph*(C^B^_London_) and CG^S^_London_ = *biograph*(C^S^_London_). A graph isomorphism, a 1-to-1 mapping of the nodes in the graph CG^B^_London_ and the nodes in the graph such that adjacencies are preserved, can then be computed, if it exists, using the *isomorphism* function in MATLAB. That is, the statement [*Isomorphic*, *Map*] = *isomorphism*(CG^B^_London_, CG^S^_London_) is used, which returns logical 1 (true) if the two N-by-N adjacency matrices extracted from biograph objects CG_London_ and CG’_London_ are isomorphic graphs and logical 0 (false) otherwise. When the Boolean value *Isomorphic* is true, *Map* is a row vector containing the node indices that map from CG^B^_London_ to CG^S^_London_. When *Isomorphic* is false, the worst-case time complexity is O(N!), where N is the number of nodes. When *Isomorphic* is true, the row vector *Map* must also be checked to see if the labeled nodes in the one structure are mapped to their correspondingly labeled nodes in the other structure. Without that, it is merely a structural isomorphism without mapping that has been detected, which is of limited value in comparing brain and perceptual data.

Isomorphism between the brain and perceptual data in fact lies simply in the two connection matrices being identical and this identity can be determined by a straightforward element-by-element comparison of the two connection matrices; for example, for *London*, an element-by-element comparison is made of CG^B^_London_ for the brain data and CG^S^_London_ for the semantic data. Failing isomorphism, which in current experimental situations is unlikely, it is useful to know what other structural similarities may hold for the brain and semantic data. These are best described in graph-theoretic terms. For two graphs G1 and G2 we would like to know what is the largest induced subgraph of G1 isomorphic to a subgraph of G2. However, this maximum common subgraph isomorphism problem is an NP-hard optimization problem. The associated decision problem, namely, given G1, G2 and an integer k, deciding if G1 contains a subgraph of at least k edges isomorphic to a subgraph of G2, is NP-complete, with no known polynomial-time solution guaranteed to work in general [46]. However, the importance of these problems to bioinformatics has given rise to other ways of approaching the problem – for example, by finding cliques [47] – and such algorithms may have application to the analysis of brain data.

What can be computed when isomorphism fails is a topological sort of each connection graph, that is, an ordering of the graph’s nodes such that if there is an edge from node x to node y, then y appears after x in the ordering. These topological sorts are exactly the partial orders of the relational structures (A, R’) presented in Section 2.2. The intersection of these topological sorts reveals the structural similarity between the language and the brain data. In general, the node order produced by a topological sort is not unique. Specifically, in the case of the brain or semantic data, every tie in the similarity differences for a given word gives rise to two distinct orderings. There is no efficient way in general to find all topological sorts; it is an NP-complete problem. However, for directed acyclic graphs it can be solved in polynomial time and a simple brute-force generation of all topological sorts is easily done for the limited number of words at play at any time in sentence understanding. Once all topological sorts are available for the brain and semantic data, the common orderings of maximal length can be extracted, also in polynomial time. These computations are easily implemented in MATLAB using its *topoorder* function. Returning to the brain and semantic data for *London*, we compute Ord^B^_London_ = *topoorder*(CG^B^_London_) and Ord^S^_London_ = *topoorder*(CG^S^_London_). We then find the longest common subsequence of Ord^B^_London_ and Ord^S^_London_. Suppose it has length L. All subsequences of this maximal length L that are common to both Ord^B^_London_ and Ord^S^_London_ jointly constitute the similarity between the brain and the semantic data.

### 2.6 Evaluating Structural Similarity

Comparing Figure 7 and Figure 8 and the Spearman rank correlation coefficients associated with these figures, it is hard to form a clear judgment of which model of semantic similarity has a better structural fit with the brain data. However, only one single-trial classification of the brain data produced the results given in these figures and the invariant partial orders were for *London* only. If the results for all the words are examined over a sufficiently large number of single-trial classifications, a different picture emerges.

We therefore performed the following computations. For each of the nine participants and for each of four sets of 10 words (three city names, three country names, four relative location words), we computed 60 single-trial classifications of the brain data using random resampling with replacement. For half of these classifications we found the partial orders that were significantly highly correlated (rho = .6485, p<0.05) and invariant with respect to the WordNet-based semantic data. We did the same for the LSA data. By using the single set of observations (the brain data) repeatedly we were engaging in a form of bootstrapping. That is, we were estimating the property of interest – the conditional probability densities – using an approximating distribution. The assumption behind this approach is that the observed data for each participant are from a population of identically distributed and independent sets of such observations.

We used four sets of words to ensure that every word appeared at least once in our computations. The sets were {*London, Moscow, Paris, north, south, east, west, Germany, Poland, Russia*}; {*Paris, Vienna, Athens, north, south, east, west, Italy, Spain, Austria*}; {*Berlin, Rome, Warsaw, north, south, east, west, France, Greece, Poland*}; and {*Madrid, Rome, Vienna, north, south, east, west, Spain, Italy, Austria*}.

**Figure 12:**
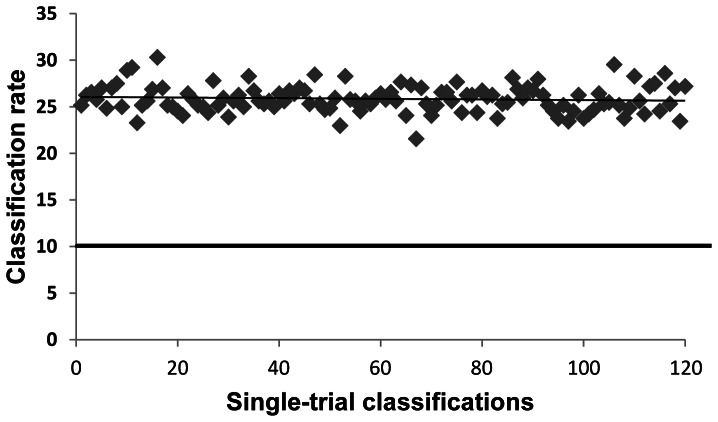
Single-trial classification rates over 120 trials for the set of words {*London, Moscow, Paris, north, south, east, west, Germany, Poland, Russia*}. 640 samples (S18) classified into 10 classes. Chance accuracy is shown by the thick solid line.

**Figure 13:**
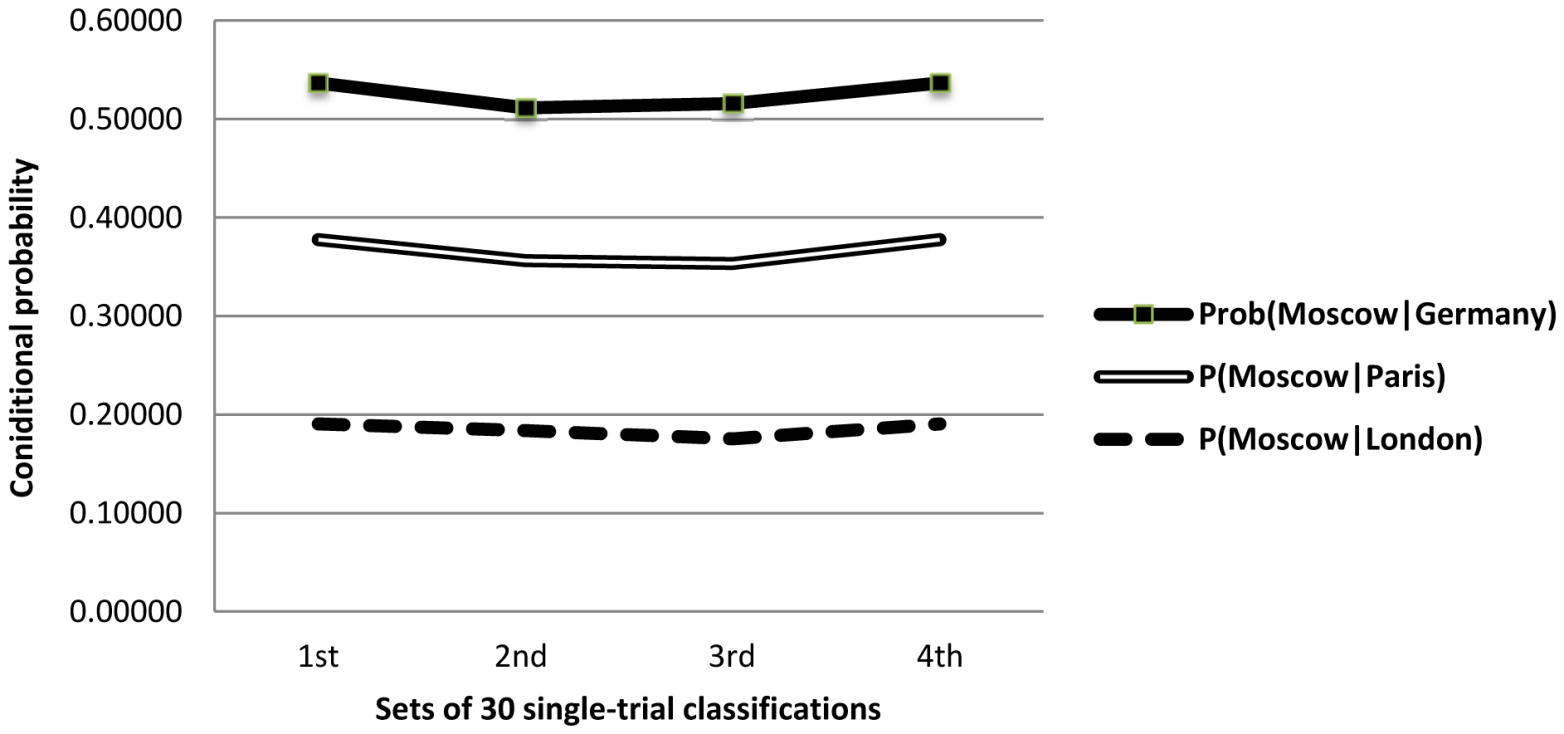
Probability that a test sample is classified as *Moscow* given that it is *London* (dash), *Paris* (double) or *Germany* (solid) over 4 sets of 30 single-trial classifications (S18) of the words {*London, Moscow, Paris, north, south, east, west, Germany, Poland, Russia*}.

**Figure 14:**
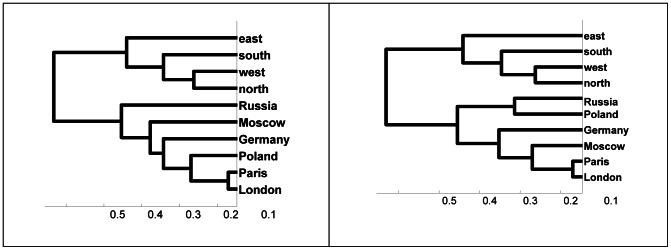
Hierarchical cluster trees computed from the accumulated average conditional probability density estimates obtained after 22 and 23 classifications of 640 brain wave samples (S18) for {*London, Moscow, Paris, north, south, east, west, Germany, Poland, Russia* **}.**

**Figure 15:**
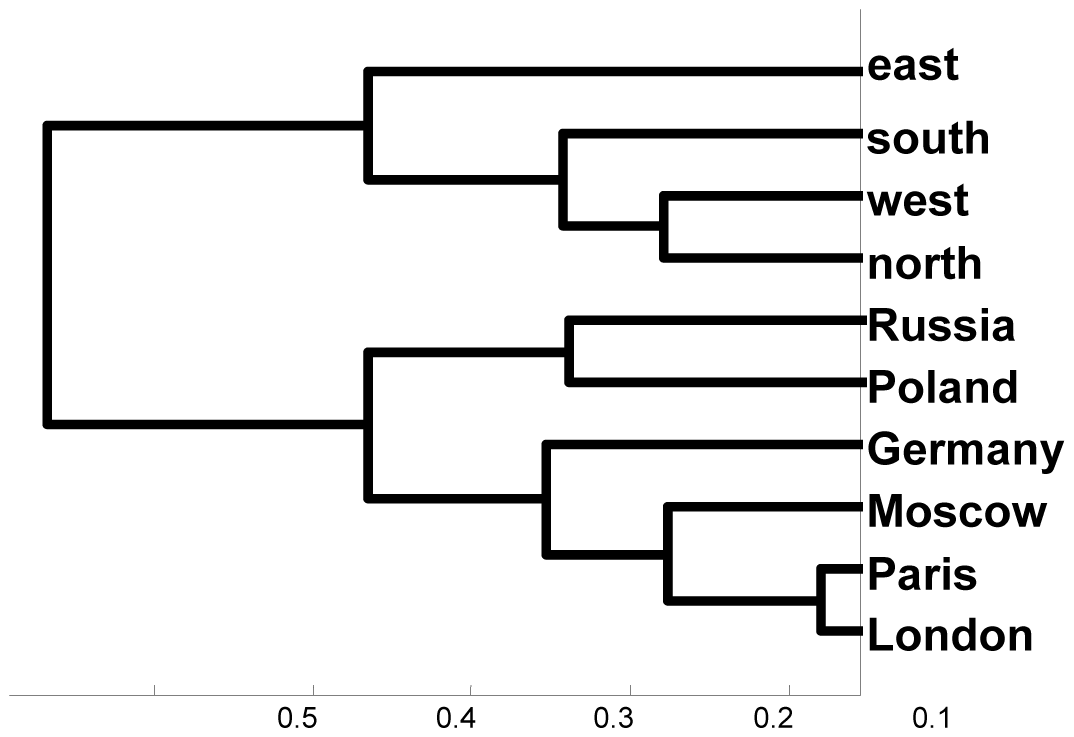
Hierarchical cluster tree computed from the accumulated conditional probability density estimates obtained after 2 classifications of 640 brain wave samples (S18) for {*London, Moscow, Paris, north, south, east, west, Germany, Poland, Russia*}. For all successive runs, the accumulated similarity tree did not change.

The average classification percent rate overall was 20.81. The average classification rates for individual participants were 16.76, 17.27, 16.97, 18.68, 16.26, 25.41, 24.69, 19.83, and 31.5. The average classification rates obtained for each of the four sets of words over the nine participants were 21.13, 21.14, 20.48, and 20.54. All rates are well above chance (10%). Furthermore, these results are stable, as illustrated in Figure 12, which presents the results of 120 single-trial classifications of the sample ten geography words for S18. These repeated computations produced consistent classification rates in the range 25% to 29%, with a mean classification rate of around 24.5%, p<10E−10. We also examined specific conditional probabilities obtained over a set of 120 single-trial classifications for four sets of 30 single-trial classifications at a time. Figure 13 shows for each set of 30 single-trial classifications (using S18) the probability that a test sample is classified as belonging to the set *Moscow* when the sample actually belongs to the set *London* (dashed line), *Paris* (double line), or *Germany* (solid line). These conditional probability estimates show similar constancy over time. As another test of the stability of the successive single-trial classifications, we computed the cumulative averages of the conditional probability estimates over two sets of 60 single-trial classifications and examined the series of similarity trees generated from these conditional probability estimates. For the first set of 60 classifications, from the 7^th^ to the 23^rd^ classification the similarity tree switched between the two configurations (left and right) in Figure 14. In both trees, *Paris* and *London* are closely coupled and *east*, *south*, *west*, and *north* are grouped identically. In the tree on the left, *Russia*, *Moscow*, *Germany* and *Poland* are intermingled. In the tree on the right, *Germany’s* grouping with the cities is the only anomaly. After the 23^rd^ classification the cumulative tree remained the same as the tree on the right. This similarity tree in fact first emerged on the 2^nd^ single-trial classification. For the second set of 60 single-trial classifications, the stable configuration emerged after the 2^nd^ classification, shown in Figure 15, and never changed.

## Results

**Figure 16:**
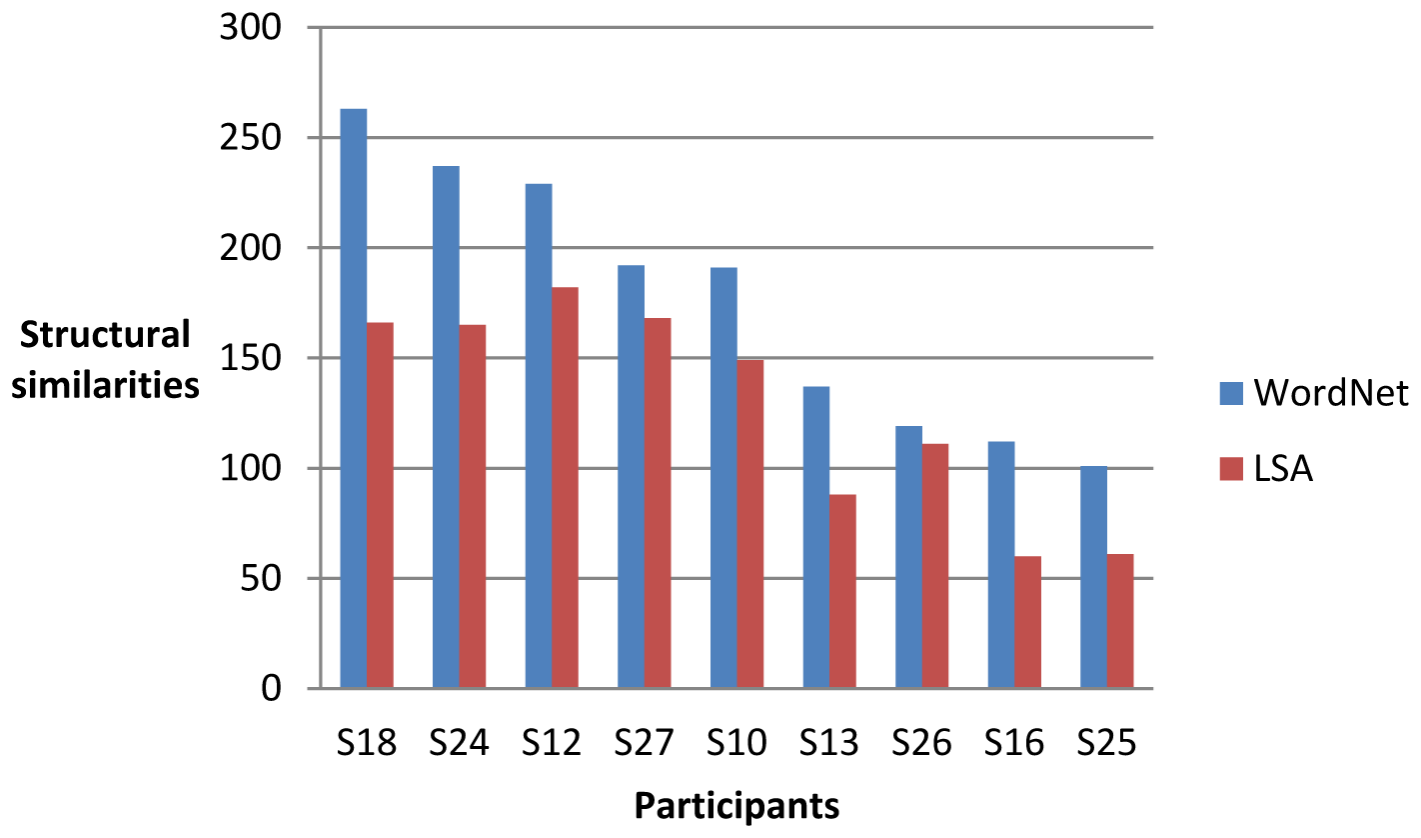
Significant structural similarities (partial orders of similarity differences invariant between the brain data and the linguistic data) for the set of words {*London, Moscow, Paris, north, south, east, west, Germany, Poland, Russia*} from 60 single-trial classifications for each participant.

**Figure 17:**
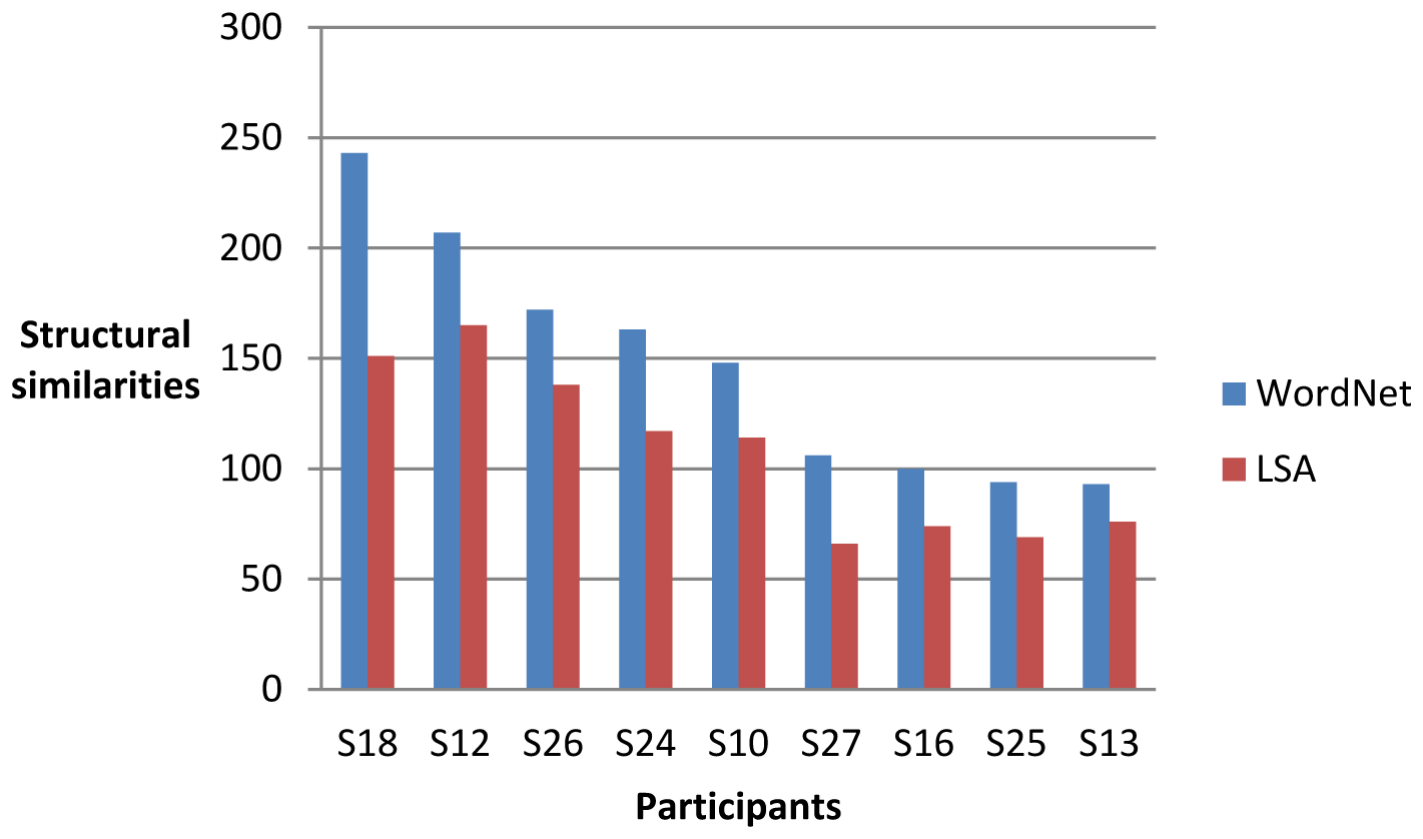
Significant structural similarities (partial orders of similarity differences invariant between the brain data and the linguistic data) for the set of words {*Paris, Vienna, Athens, north, south, east, west, Italy, Spain, Austria*} from 60 single-trial classifications for each participant.

**Figure 18:**
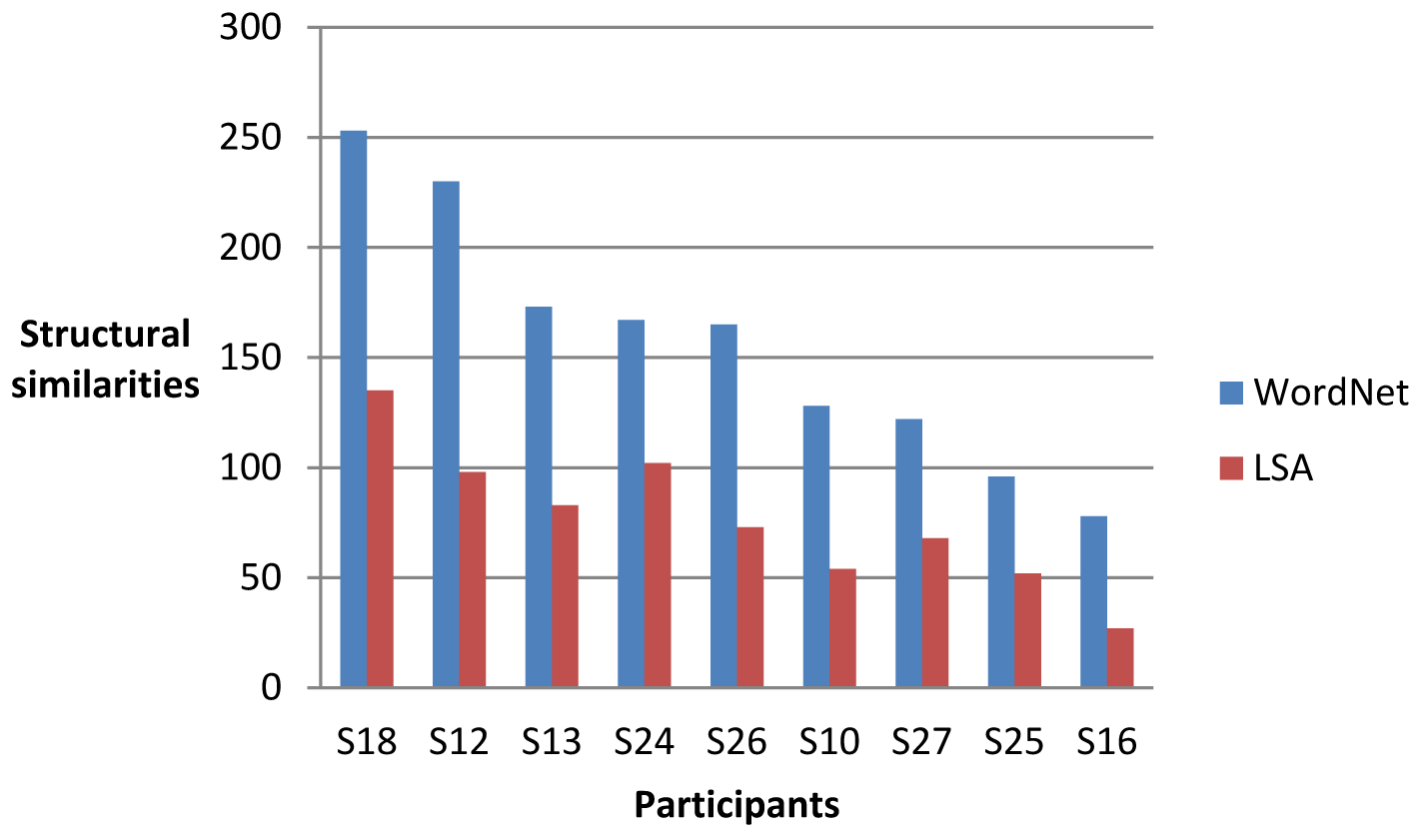
Significant structural similarities (partial orders of similarity differences invariant between the brain data and the linguistic data) for the set of words {*Berlin, Rome, Warsaw, north, south, east, west, France, Greece, Poland*} from 60 single-trial classifications for each participant.

**Figure 19:**
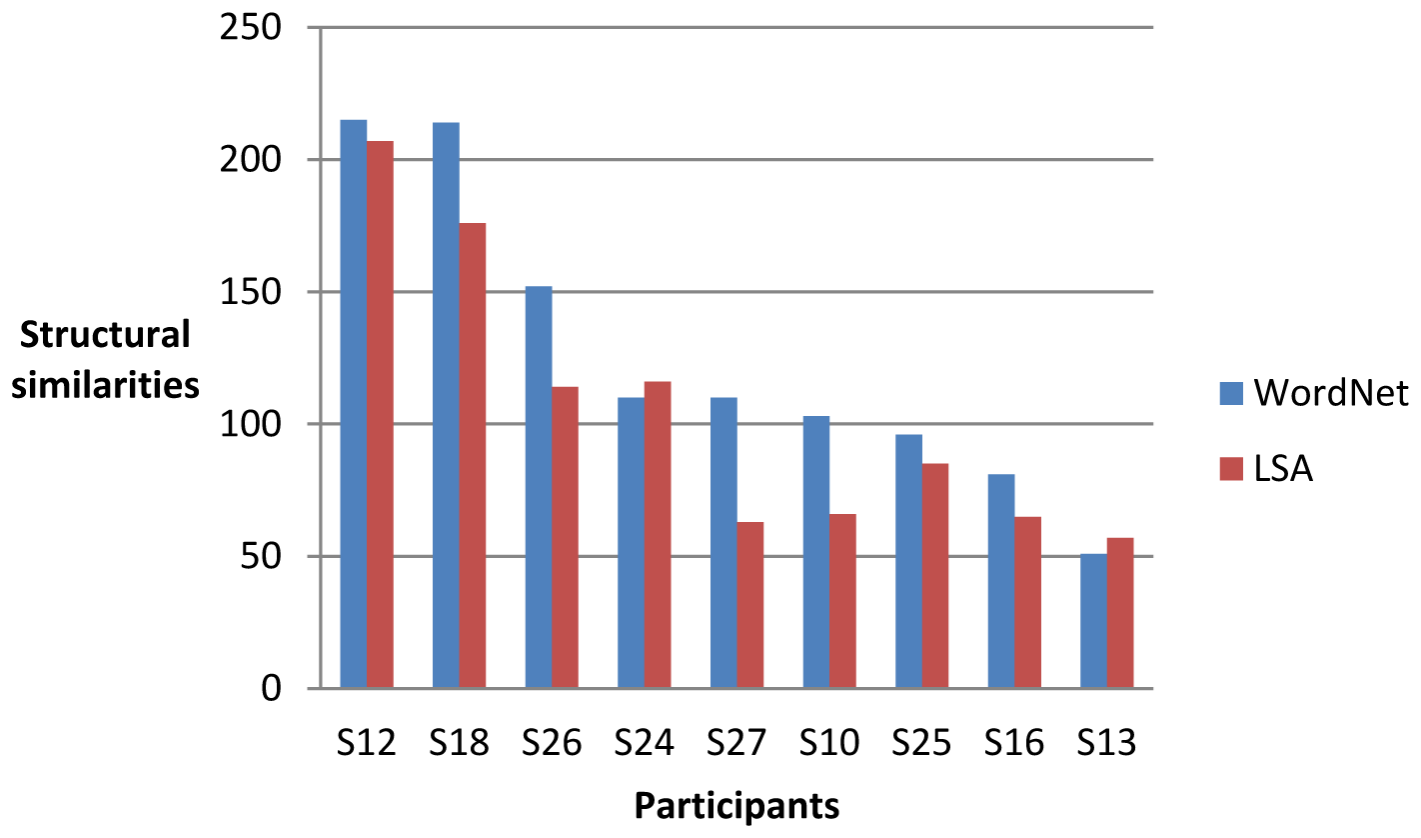
Significant structural similarities (partial orders of similarity differences invariant between the brain data and the linguistic data) for the set of words {*Madrid, Rome, Vienna, north, south, east, west, Spain, Italy, Austria*} from 60 single-trial classifications for each participant.

For each of the nine participants (identified as S10, S12, S13, S16, S18, S24, S25, 26 and S27) and each of four sets of words, we computed two sets of 30 single-trial classifications of the brain data as described above. One set of results from the brain data for each participant was compared with the linguistic data derived from WordNet and the other LSA. We then assessed the degree of structural similarity between the brain data and the linguistic data. The maximum number of significant invariant partial orders that could be obtained in each set of single-trial classifications was 300 (30 classifications of 10 words). Figure 16, Figure 17, Figure 18 and Figure 19 give the results for WordNet and LSA for each participant for the four sets of words. A higher number of significant structural similarities were found between the brain data and the WordNet data than the LSA data. This pattern held for all but two of the 72 experimental runs. It is interesting to note that the difference between the number of structural similarities for WordNet and LSA was smallest for the set of words that had for each city the country of which it was the capital, namely the set {*Madrid, Rome, Vienna, north, south, east, west, Spain, Italy, Austria*}.

## Discussion

We start with some possible objections to the results presented in this paper. First is the consideration that some other property of the stimuli co-varies with the words of interest and is responsible for the recognition rates and confusion matrices. Two possible confounding variables are the length of the words and the number of syllables. The relative location words *north*, *south*, *east* and *west* are all one syllable and shorter than most of the city and country names, only a few of which are one syllable. With the modified sample lengths as described in section 2.1, however, the data samples for these words correspond roughly to the two-word and two-syllable utterances *north of*, *south of, east of, and west of*. The three-syllable names – *Vienna, Italy, Germany and Austria* – are misclassified relative to the other words at approximately the same rate as the two-syllable words and so their possible confounding effect is taken to be minor for this study. A third possible confounding variable is that of grammatical class, which is typically highly correlated with word meaning. In our geography sentences, the grammatical roles of the country and city names are different from those of the relative location terms (*east*, *west*, *north*, and *south*). It is not immediately clear to what extent, if any, our analysis has been affected by grammatical class. It is significant, however, that the city names and country names are clearly differentiated despite being from the same grammatical class. Furthermore, any confounding effect would be present in the structural similarity evaluation for both semantic models and so would not invalidate the comparative conclusion we have reached.

Another possible objection to the results presented in this paper is that the recognition rates and confusion matrices reflect the probabilities of each word’s occurrence in the sentences. That is, *north* and *south* and *east* and *west* are confused with each other because of their expected occurrences in their given positions in the sentences. Similarly for the country names and city names. In this case, a probabilistic semantic grammar could perhaps explain the confusion matrices, without any recourse to positing WordNet or LSA as an underlying semantic model. The notion of a word’s expected appearance at a particular point in a sentence is related to the notion of cloze probability, which is defined as the probability of a specific word completing a particular part of a sentence. Kutas and Hillyard reported that the N400 ERP amplitude for a word has a nearly inverse linear relationship with its cloze probability [32]. The higher the expectation that a word will appear in a given context, the lower its N400 is relative to words with lower expectation. Generally for nouns, verbs, adjectives, and adverbs, the N400 is also lower for words appearing later in a sentence compared to earlier. Further experimentation is needed to investigate the N400 components for categories of words and their occurrences in different parts of our geography sentences.

The new methods introduced in this paper have been illustrated using two different semantic models. The stronger structural similarity between the brain data and the WordNet-derived data seems to support the claim that during sentence comprehension the representation of words in the brain has a WordNet-like quality. To evaluate what that may mean, we have to ask what the crucial differences are between the two semantic models and the measures of similarity afforded by each.

Wordnet was developed using human judgments about the meanings of the words and relations between them. WordNet encodes fine-grained sense distinctions, grouping two words into the same synonym set if and only if in some context they can be substituted for each other without changing the truth value of the proposition. WordNet has been criticized for encoding sense distinctions that some think are too fine-grained even for humans. For example, consider the following two senses of *north* used in our work:

(n) north#3, due north#1, northward#1, N#2 (the cardinal compass point that is at 0 or 360 degrees).

(n) north#5 (the direction corresponding to the northward cardinal compass point).

However, a point is fundamentally different from a direction; the two senses cannot be substituted for each other in all contexts. There is an irreducible distinction between the two senses, even if very few people could articulate it and it is most likely seldom at work in people’s uses of the word. WordNet has been particularly successfully applied computationally in word-sense disambiguation, that is, in determining which of the candidate senses of a word is most appropriate in a given context. WordNet senses have been aggregated in some applications, as indeed they were in our work here. In this way the fine-grained distinctions prevail in the network of sense connections present in WordNet, but the distinctions do not dominate.

The strongest criticism of WordNet has been related to its inadequacies as an ontology [48]. For example, WordNet does not distinguish between one thing being a subtype as opposed to an instance of another thing, as with the Bronze Age, the Iron Age and so on being instances, not subtypes, of time periods. Its taxonomic organization is imperfect. Wordnet also has limited coverage of specialized vocabularies. Another criticism is that WordNet gives obscure senses the same prominence as more frequent senses, as with Paris the city and Paris the character in literature. The last criticism has no impact on our results as only relevant senses were used. As to the ontological criticism, it would be a surprise to discover that when it came to ordinary people, brain representations met strict logical conditions. Only further experimentation will tell.

Since Lund and Burgess, distributional corpus-based models, ranging from simple co-occurrence vectors to probabilistic topic-based approaches such as LDA, have increasingly gained acceptance [49]. LSA and other distributional models do not in any obvious way connect all words and all senses of all words via semantic relations. Nor do they systematically represent fine-grained distinctions between different words and different senses of a word. There are, however, many ways in which one distributional model differs from another, some of which were discussed in the Introduction. The size and content of the corpus vary, as do the normalization techniques, dimensionality reduction strategies, and measures of similarity. When we point out the greater structual similarity of the WordNet-derived data with the brain data over the LSA-derived data, our conclusions have to be circumscribed. LSA cannot function as a proxy for all distributional models. What we can say is that the WordNet model seems to have an advantage over corpus-based distributional models, particularly when it comes to looking at the network of relations between words in a sentence comprehension task.

One important point about distributional models is that they require validation. Typically, validation entails seeing how well a given model correlates with the semantic judgments of native speakers. When speakers’ predictions diverge from a model, questions arise as to whether that divergence points to limitations in the model or inadequacies in how those judgments were solicited, whether they were fine-grained enough, for example. Judgments obtained directly from neural activity would potentially be more accurate, an approach considered in the work of Devereux and others discussed in the Introduction [21].

At this point it is useful to explore what differentiates our work from that of Kriegeskort *et al.* [50]. In both cases, representational models of brain activity are constructed and compared with representational models of some aspect of the experimental situation, whether the stimuli that elicited the neural activity or the behavior exhibited in response to the stimuli, for example. There are, however, important differences between the Kriegeskorte approach and ours. Kriegeskorte and others, two relational structures are compiled, one a representation of brain activity and the other a representation of some aspect of the experimental setup [50]. Their relational structures are in fact representational dissimilarity matrices, one constructed for measures of neural activity and the other for the stimuli eliciting the neural activity or the behavior accompanying the neural activity. Dissimilarity matrices, like our conditional probability density estimates and semantic similarity matrices, offer a general way to characterize the information carried by a given representation, whether it is a representation of the brain activity or a representation of the stimuli, such as words in their sentential context. But the methods they use to compare their representational dissimilarity matrices differ from the methods we use to compare the invariant partial orders of our representational structures. Kriegeskorte and others argue against a linear match between dissimilarity matrices; instead, like us, they use the Spearman rank correlation coefficient [50]. However, we go a fundamentally important step further and, for those similarity matrices that are significantly strongly correlated, we construct the partial orders that are invariant with respect to the brain data and the experimental data. These invariant structures give a detailed assessment of where the similarities between the brain data and the linguistic data actually lie. These invariant structures will be invaluable in future work, such as when we take into account more information in the experimental setup, for example, the participants’ responses – namely whether they judged a statement true or false and whether they were right or wrong. Formulating and testing complex hypotheses about brain activity become possible with the methods presented in this paper.

In considering how the brain as a system processes language, the idea of an associative network has long held appeal. The work on associative networks by Collins and Loftus [51] and the investigation of the semantics of associative networks by Woods [52] are early contributions. Associative networks for ambiguity resolution have received a lot of attention, beginning with Hirst [53]. Associative networks for information retrieval and web search have gained increasing attention, starting with Crestani [54]. Activation and spreading activation, the principal operations proposed for associative networks, have been characterized in various ways over time, but no specific method or algorithm has yet emerged as definitive. What is still missing from discussions of associative networks in language processing is a coherent, general account of how associative networks are used in complex tasks such as answering a question or determining the truth or falsity of an assertion. In this paper, associative networks such as those in Figure 10 and Figure 11 have been presented as the brain’s dynamic representation of a set of words during the response to a sentence such as *Rome is not the capital of Spain*. Such associative networks are not static, but are configurations that change under different stimulus conditions and under the operations of activation and spreading activation. However, these changes are not unconstrained. The associative networks are approximately invariant across language users and across different occasions on which the words are used. What this paper suggests is that the associative networks underlying sentence comprehension, particularly for the task of determining the truth or falsity of an assertion, are possibly more like the associative networks of WordNet than those obtained by statistical methods from large corpora.

WordNet is far from the end of the story, however. Binary, ternary and quaternary relations are generally missing from WordNet, but are common in ordinary language. The sentence *Rome is a capital* may be represented with some ease using WordNet and its *is_a* relation, and the truth or falsity of the assertion determined by the simple test: is there an *is_a* relation between *Rome* and *capital*? But *Rome is the capital of Italy*, expressing a binary relation, has no straightforward representation in WordNet, and none that permits its truth or falsity to be determined directly. Similarly for ternary relations such as those expressed in the sentence *Sue gave Bill a book* or, using our geography words, *Paris is more north of Rome than west of Berlin*. Associative networks that include binary and higher-order relations, along with definitions of activation and spreading activation for each order, are still needed. Large enough or focused enough corpora could potentially provide such associations We noted earlier the work of Kelly and others who used parsed data to identify three-part associations involving the well-known taxonomic relations of *is-a* and *part-of* in Wikipedia and the British National Corpus [20]. The recognition of higher-order relations, such as those in *Paris is the capital of France* and *Paris is further west of Moscow than Berlin is,* would be a natural progression from that work. The methods of relative structural similarity developed in this paper can be used to assess the extent to which any network of semantic relations is structurally similar to brain data collected during a language task.

## Supporting Information

Figure S1 a − j: WordNet-based and LSA-based semantic similarities and brain conditional probability estimates for (a) *London*; (b) *Moscow*; (c) *Paris*; (d) *north*; (e) *south*; (f) *east*; (g) *west*; (h) *Germany*; (i) *Poland*; and (j) *Russia,* relative to *London*, *Moscow*, *Paris*, *north*, *south*, *east*, *west*, *Germany*, *Poland*, and *Russia*. Data taken from Figure 1, Figure 2, and Figure 4.
(TIFF)

Material S1 Trigger sentences and timings. Table S1: Timings for trigger sentences. Table S2: Timings for the presentation of the individual words in each of the 48 trigger sentences.
(DOCX)

Material S2 WordNet entries.
(DOCX)

Material S3 Five Wordnet-based measures of similarity.
(DOCX)
