# Supplementary figures and images for: Structural Similarities between Brain and Linguistic Data Provide Evidence of Semantic Relations in the Brain
Source: PLoS One. 2013 Jun 14;8(6):e65366. doi: 10.1371/journal.pone.0065366 (PMC3682999; doi:10.1371/journal.pone.0065366)

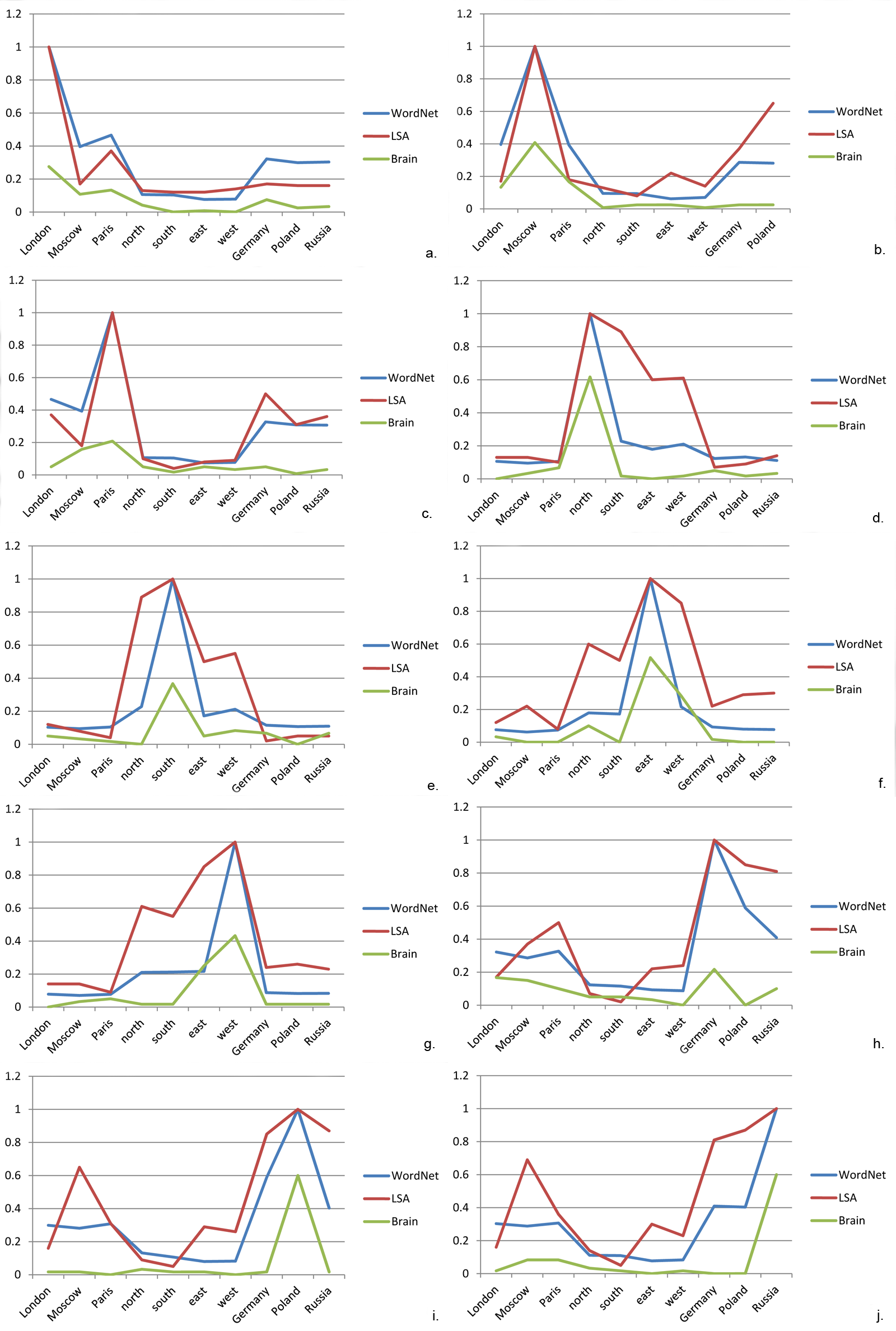

Supplement: Figure S1 — a − j: WordNet-based and LSA-based semantic similarities and brain conditional probability estimates for (a) London; (b) Moscow; (c) Paris; (d) north; (e) south; (f) east; (g) west; (h) Germany; (i) Poland; and (j) Russia, relative to London, Moscow, Paris, north, south, east, west, Germany, Poland, and Russia. Data taken from Figure 1, Figure 2, and Figure 4. (TIFF) [file pone.0065366.s001.tiff]
