# Supplementary material for: Structural Similarities between Brain and Linguistic Data Provide Evidence of Semantic Relations in the Brain
Source: PLoS One. 2013 Jun 14;8(6):e65366. doi: 10.1371/journal.pone.0065366 (PMC3682999; doi:10.1371/journal.pone.0065366)
Supplement: Material S1 — Trigger sentences and timings. Table S1: Timings for trigger sentences. Table S2: Timings for the presentation of the individual words in each of the 48 trigger sentences. (DOCX) [file pone.0065366.s002.docx]

## S1 – Trigger sentences and timings

|  |  | | seconds | ms |  |
| --- | --- | --- | --- | --- | --- |
|  | Sentence Length | Max | 2.59 | 2590 |  |
|  |  | Min | 1.35 | 1350 |  |
|  | Onset to onset | | 4.5 | 4500 |  |

###### Table S1: Timings for trigger sentences

|  |  | 1 |
| --- | --- | --- |
| The | 0.047 | 0.186 |
| capital | 0.22 | 0.831 |
| of | 0.864 | 1.096 |
| Italy | 1.099 | 1.623 |
| is | 1.631 | 1.824 |
| Paris | 1.88 | 2.452 |
|  |  | 2 |
| London | 0.062 | 0.648 |
| is | 0.684 | 0.887 |
| not | 0.891 | 1.188 |
| the | 1.247 | 1.406 |
| capital | 1.414 | 1.833 |
| of | 1.84 | 1.975 |
| Poland | 2.05 | 2.561 |
|  |  | 3 |
| The | 0.025 | 0.161 |
| largest | 0.175 | 0.703 |
| city | 0.725 | 1.105 |
| of | 1.11 | 1.247 |
| France | 1.283 | 1.682 |
| is | 1.682 | 1.786 |
| not | 1.791 | 2.006 |
| Berlin | 2.059 | 2.481 |
|  |  | 4 |
| Warsaw | 0.041 | 0.669 |
| is | 0.711 | 0.848 |
| not | 0.848 | 1.09 |
| the | 1.124 | 1.238 |
| largest | 1.252 | 1.617 |
| city | 1.631 | 2.008 |
| of | 2.041 | 2.203 |
| Russia | 2.221 | 2.633 |
|  |  | 5 |
| Moscow | 0.027 | 0.708 |
| is | 0.817 | 1.088 |
| east | 1.16 | 1.612 |
| of | 1.634 | 1.821 |
| Warsaw | 1.838 | 2.368 |
|  |  | 6 |
| Rome | 0.039 | 0.354 |
| is | 0.365 | 0.532 |
| north | 0.541 | 0.811 |
| of | 0.817 | 0.917 |
| London | 0.937 | 1.386 |
|  |  | 7 |
| Paris | 0.027 | 0.479 |
| is | 0.485 | 0.577 |
| not | 0.591 | 0.808 |
| west | 0.845 | 1.104 |
| of | 1.121 | 1.235 |
| Berlin | 1.283 | 1.729 |
|  |  | 8 |
| Rome | 0.03 | 0.51 |
| is | 0.53 | 0.714 |
| not | 0.719 | 0.976 |
| south | 0.995 | 1.442 |
| of | 1.551 | 1.665 |
| Moscow | 1.676 | 2.248 |
|  |  | 9 |
| The | 0.039 | 0.161 |
| capital | 0.198 | 0.658 |
| of | 0.664 | 0.8 |
| Germany | 0.811 | 1.308 |
| is | 1.436 | 1.618 |
| Warsaw | 1.629 | 2.162 |
|  |  | 10 |
| Moscow | 0.033 | 0.683 |
| is | 0.716 | 0.864 |
| not | 0.875 | 1.121 |
| the | 1.166 | 1.285 |
| Capital | 1.316 | 1.846 |
| of | 1.93 | 2.103 |
| Russia | 2.117 | 2.516 |
|  |  | 11 |
| The | 0.036 | 0.117 |
| largest | 0.139 | 0.622 |
| city | 0.627 | 0.873 |
| of | 0.906 | 0.965 |
| Italy | 1.015 | 1.403 |
| is | 1.556 | 1.729 |
| not | 1.751 | 1.952 |
| Rome | 2.025 | 2.373 |
|  |  | 12 |
| London | 0.027 | 0.521 |
| is | 0.543 | 0.7 |
| not | 0.714 | 0.979 |
| the | 1.007 | 1.118 |
| largest | 1.129 | 1.609 |
| city | 1.587 | 1.849 |
| of | 1.857 | 1.997 |
| France | 2.014 | 2.53 |
|  |  | 13 |
| Paris | 0.027 | 0.596 |
| is | 0.661 | 0.945 |
| east | 0.984 | 1.35 |
| of | 1.355 | 1.459 |
| Berlin | 1.461 | 1.897 |
|  |  | 14 |
| Moscow | 0.027 | 0.672 |
| is | 0.792 | 0.959 |
| North | 0.968 | 1.486 |
| of | 1.503 | 1.662 |
| Paris | 1.729 | 2.29 |
|  |  | 15 |
| Warsaw | 0.019 | 0.675 |
| is | 0.783 | 0.973 |
| not | 0.979 | 1.286 |
| west | 1.313 | 1.807 |
| of | 1.866 | 2.014 |
| London | 2.019 | 2.482 |
|  |  | 16 |
| Berlin | 0.03 | 0.677 |
| is | 0.769 | 0.948 |
| not | 0.951 | 1.163 |
| south | 1.224 | 1.835 |
| of | 1.902 | 2.072 |
| Rome | 2.092 | 2.552 |
|  |  | 17 |
| The | 0.013 | 0.145 |
| capital | 0.186 | 0.691 |
| of | 0.728 | 0.967 |
| Italy | 0.976 | 1.461 |
| is | 1.55 | 1.729 |
| not | 1.74 | 1.974 |
| Berlin | 2.002 | 2.463 |
|  |  | 18 |
| Warsaw | 0.025 | 0.633 |
| is | 0.728 | 0.917 |
| the | 0.931 | 1.029 |
| capital | 1.059 | 1.609 |
| of | 1.676 | 1.91 |
| France | 1.921 | 2.468 |
|  |  | 19 |
| The | 0.019 | 0.164 |
| largest | 0.17 | 0.663 |
| city | 0.666 | 0.848 |
| of | 0.853 | 0.976 |
| Germany | 1.02 | 1.539 |
| is | 1.687 | 1.827 |
| Berlin | 1.902 | 2.29 |
|  |  | 20 |
| London | 0.016 | 0.563 |
| is | 0.694 | 0.811 |
| the | 0.834 | 0.928 |
| largest | 1.023 | 1.545 |
| city | 1.548 | 1.757 |
| of | 1.762 | 1.854 |
| Russia | 1.905 | 2.32 |
|  |  | 21 |
| Moscow | 0.025 | 0.764 |
| is | 0.875 | 1.054 |
| not | 1.118 | 1.545 |
| east | 1.626 | 1.863 |
| of | 1.919 | 1.986 |
| Paris | 2.086 | 2.616 |
|  |  | 22 |
| Rome | 0.033 | 0.557 |
| is | 0.652 | 0.834 |
| not | 0.845 | 1.107 |
| north | 1.124 | 1.403 |
| of | 1.425 | 1.537 |
| Warsaw | 1.584 | 2.114 |
|  |  | 23 |
| London | 0.03 | 0.56 |
| is | 0.697 | 0.923 |
| west | 0.954 | 1.414 |
| of | 1.489 | 1.623 |
| Moscow | 1.679 | 2.218 |
|  |  | 24 |
| Paris | 0.025 | 0.571 |
| is | 0.65 | 0.864 |
| south | 0.892 | 1.364 |
| of | 1.389 | 1.52 |
| Rome | 1.581 | 1.869 |
|  |  | 25 |
| Vienna | 0.053 | 0.615 |
| is | 0.682 | 0.86 |
| south | 0.862 | 1.187 |
| of | 1.204 | 1.313 |
| London | 1.317 | 1.671 |
|  |  | 26 |
| Madrid | 0.057 | 0.652 |
| is | 0.701 | 0.871 |
| not | 0.874 | 1.114 |
| west | 1.146 | 1.416 |
| of | 1.448 | 1.531 |
| Athens | 1.591 | 2.161 |
|  |  | 27 |
| Berlin | 0.051 | 0.694 |
| is | 0.82 | 0.964 |
| not | 0.974 | 1.154 |
| the | 1.181 | 1.298 |
| capital | 1.315 | 1.681 |
| of | 1.686 | 1.777 |
| Greece | 1.837 | 2.324 |
|  |  | 28 |
| Athens | 0.096 | 0.646 |
| is | 0.724 | 0.91 |
| the | 0.913 | 1.02 |
| largest | 1.041 | 1.562 |
| city | 1.565 | 1.745 |
| of | 1.756 | 1.829 |
| Spain | 1.834 | 2.326 |
|  |  | 29 |
| Moscow | 0.042 | 0.682 |
| is | 0.829 | 0.946 |
| west | 0.974 | 1.27 |
| of | 1.32 | 1.395 |
| Vienna | 1.439 | 1.858 |
|  |  | 30 |
| The | 0.045 | 0.178 |
| largest | 0.189 | 0.711 |
| city | 0.719 | 0.889 |
| of | 0.897 | 1.046 |
| Poland | 1.122 | 1.66 |
| is | 1.687 | 1.858 |
| Madrid | 1.868 | 2.509 |
|  |  | 31 |
| Paris | 0.051 | 0.635 |
| is | 0.752 | 0.898 |
| not | 0.903 | 1.117 |
| south | 1.133 | 1.392 |
| of | 1.401 | 1.483 |
| Athens | 1.585 | 2.17 |
|  |  | 32 |
| The | 0.019 | 0.134 |
| capital | 0.158 | 0.578 |
| of | 0.585 | 0.677 |
| Russia | 0.709 | 1.136 |
| is | 1.245 | 1.373 |
| Madrid | 1.386 | 1.967 |
|  |  | 33 |
| The | 0.018 | 0.123 |
| largest | 0.139 | 0.62 |
| city | 0.623 | 0.792 |
| of | 0.803 | 0.881 |
| Austria | 0.905 | 1.426 |
| is | 1.555 | 1.733 |
| not | 1.746 | 1.961 |
| Warsaw | 2.069 | 2.544 |
|  |  | 34 |
| Warsaw | 0.041 | 0.666 |
| is | 0.838 | 1.001 |
| north | 1.029 | 1.468 |
| of | 1.505 | 1.636 |
| Rome | 1.659 | 2.072 |
|  |  | 35 |
| Moscow | 0.03 | 0.679 |
| is | 0.767 | 0.941 |
| not | 0.948 | 1.19 |
| the | 1.245 | 1.363 |
| capital | 1.386 | 1.75 |
| of | 1.751 | 1.872 |
| Austria | 1.879 | 2.357 |
|  |  | 36 |
| The | 0.023 | 0.129 |
| capital | 0.187 | 0.57 |
| of | 0.583 | 0.667 |
| Spain | 0.67 | 1.293 |
| is | 1.38 | 1.528 |
| not | 1.541 | 1.797 |
| Paris | 1.897 | 2.433 |
|  |  | 37 |
| Greece | 0.046 | 0.48 |
| is | 0.585 | 0.709 |
| west | 0.78 | 1.09 |
| of | 1.093 | 1.164 |
| Italy | 1.206 | 1.552 |
|  |  | 38 |
| Poland | 0.007 | 0.528 |
| is | 0.607 | 0.775 |
| west | 0.827 | 1.109 |
| of | 1.115 | 1.294 |
| France | 1.308 | 1.815 |
|  |  | 39 |
| Austria | 0.042 | 0.676 |
| is | 0.759 | 0.949 |
| not | 0.966 | 1.207 |
| north | 1.271 | 1.523 |
| of | 1.557 | 1.664 |
| Greece | 1.73 | 2.233 |
|  |  | 40 |
| Vienna | 0.036 | 0.664 |
| is | 0.77 | 0.919 |
| not | 0.928 | 1.222 |
| east | 1.289 | 1.559 |
| of | 1.588 | 1.674 |
| Madrid | 1.756 | 2.274 |
|  |  | 41 |
| Germany | 0.028 | 0.68 |
| is | 0.758 | 0.951 |
| not | 0.967 | 1.167 |
| South | 1.182 | 1.473 |
| of | 1.481 | 1.617 |
| Spain | 1.622 | 2.126 |
|  |  | 42 |
| Athens | 0.019 | 0.669 |
| is | 0.75 | 0.839 |
| south | 0.853 | 1.208 |
| of | 1.212 | 1.273 |
| London | 1.306 | 1.706 |
|  |  | 43 |
| The | 0.034 | 0.168 |
| largest | 0.178 | 0.672 |
| city | 0.687 | 0.872 |
| of | 0.882 | 0.979 |
| Greece | 1.045 | 1.592 |
| is | 1.659 | 1.854 |
| Vienna | 1.859 | 2.346 |
|  |  | 44 |
| Moscow | 0.051 | 0.658 |
| is | 0.782 | 0.998 |
| not | 1.01 | 1.228 |
| north | 1.284 | 1.57 |
| of | 1.575 | 1.668 |
| Madrid | 1.69 | 2.292 |
|  |  | 45 |
| Austria | 0.054 | 0.624 |
| is | 0.686 | 0.881 |
| not | 0.888 | 1.12 |
| east | 1.186 | 1.474 |
| of | 1.499 | 1.576 |
| Spain | 1.635 | 2.083 |
|  |  | 46 |
| The | 0.031 | 0.161 |
| capital | 0.197 | 0.61 |
| of | 0.617 | 0.722 |
| Germany | 0.772 | 1.321 |
| is | 1.385 | 1.6 |
| Rome | 1.617 | 2.107 |
|  |  | 47 |
| Russia | 0.022 | 0.515 |
| is | 0.598 | 0.826 |
| east | 0.861 | 1.114 |
| of | 1.162 | 1.253 |
| Spain | 1.261 | 1.884 |
|  |  | 48 |
| The | 0.018 | 0.132 |
| capital | 0.176 | 0.58 |
| of | 0.588 | 0.696 |
| Poland | 0.777 | 1.285 |
| is | 1.305 | 1.487 |
| not | 1.497 | 1.759 |
| Athens | 1.787 | 2.43 |

###### Table S2: Timings for the presentation of the individual words in each of the 48 trigger sentences
