# Supplementary material for: Structural Similarities between Brain and Linguistic Data Provide Evidence of Semantic Relations in the Brain
Source: PLoS One. 2013 Jun 14;8(6):e65366. doi: 10.1371/journal.pone.0065366 (PMC3682999; doi:10.1371/journal.pone.0065366)
Supplement: Material S2 — WordNet entries. (DOCX) [file pone.0065366.s003.docx]

## S2 -- WordNet entries

All WordNet senses for the reference set of geography words {London, Moscow, Paris, north, south, east, west, Germany, Poland, Russia} are shown. Those senses not directly relevant to the geography of Europe are indented.

**London**

(n) **London#1**, Greater London#1, British capital#1, capital of the United Kingdom#1 (the capital and largest city of England; located on the Thames in southeastern England; financial and industrial and cultural center)

(n) **London#2**, Jack London#1, John Griffith Chaney#1 (United States writer of novels based on experiences in the Klondike gold rush (1876-1916))

|  |  |
| --- | --- |

**Moscow**

(n) **Moscow#1**, capital of the Russian Federation#1, Russian capital#1 (a city of central European Russia; formerly capital of both the Soviet Union and Soviet Russia; since 1991 the capital of the Russian Federation)

**Paris**

(n) **Paris#1**, City of Light#1, French capital#1, capital of France#1 (the capital and largest city of France; and international center of culture and commerce)

(n) **Paris#2**, genus Paris#1 (sometimes placed in subfamily Trilliaceae)

(n) **Paris#3** ((Greek mythology) the prince of Troy who abducted Helen from her husband Menelaus and provoked the Trojan War)

(n) **Paris#4** (a town in northeastern Texas)

**Berlin**

(n) **Berlin#1**, [German capital#1](http://wordnetweb.princeton.edu/perl/webwn?o2=&o0=1&o7=1&o5=&o1=1&o6=&o4=&o3=&s=German+capital) (capital of Germany located in eastern Germany)

(n) **Berlin#2**, [Irving Berlin#1](http://wordnetweb.princeton.edu/perl/webwn?o2=&o0=1&o7=1&o5=&o1=1&o6=&o4=&o3=&s=Irving+Berlin), [Israel Baline#1](http://wordnetweb.princeton.edu/perl/webwn?o2=&o0=1&o7=1&o5=&o1=1&o6=&o4=&o3=&s=Israel+Baline) (United States songwriter (born in Russia) who wrote more than 1500 songs and several musical comedies (1888-1989))

(n) **berlin#3** (a limousine with a glass partition between the front and back seats)

**Athens**

(n) **Athens#1**, [Athinai#1](http://wordnetweb.princeton.edu/perl/webwn?o2=&o0=1&o7=1&o5=&o1=1&o6=&o4=&o3=&s=Athinai), [capital of Greece#1](http://wordnetweb.princeton.edu/perl/webwn?o2=&o0=1&o7=1&o5=&o1=1&o6=&o4=&o3=&s=capital+of+Greece), [Greek capital#1](http://wordnetweb.princeton.edu/perl/webwn?o2=&o0=1&o7=1&o5=&o1=1&o6=&o4=&o3=&s=Greek+capital) (the capital and largest city of Greece; named after Athena (its patron goddess)) *"in the 5th century BC ancient Athens was the world's most powerful and civilized city"*

(n) **Athens#2** (a town in southeast Ohio)

(n) **Athens#3** (a university town in northeast Georgia)

**Vienna**

(n) **Vienna#1**, [Austrian capital#1](http://wordnetweb.princeton.edu/perl/webwn?o2=&o0=1&o7=1&o5=&o1=1&o6=&o4=&o3=&s=Austrian+capital), [capital of Austria#1](http://wordnetweb.princeton.edu/perl/webwn?o2=&o0=1&o7=1&o5=&o1=1&o6=&o4=&o3=&s=capital+of+Austria) (the capital and largest city of Austria; located on the Danube in northeastern Austria; was the home of Beethoven and Brahms and Haydn and Mozart and Schubert and Strauss)

[**Madrid**](http://wordnetweb.princeton.edu/perl/webwn?o2=&o0=1&o7=1&o5=&o1=1&o6=&o4=&o3=&s=Madrid&i=0&h=0#c)

(n) **Madrid#1**, [capital of Spain#1](http://wordnetweb.princeton.edu/perl/webwn?o2=&o0=1&o7=1&o5=&o1=1&o6=&o4=&o3=&s=capital+of+Spain), [Spanish capital#1](http://wordnetweb.princeton.edu/perl/webwn?o2=&o0=1&o7=1&o5=&o1=1&o6=&o4=&o3=&s=Spanish+capital) (the capital and largest city situated centrally in Spain; home of an outstanding art museum)

**Rome**

(n) **Rome#1**, [Roma#2](http://wordnetweb.princeton.edu/perl/webwn?o2=&o0=1&o7=1&o5=&o1=1&o6=&o4=&o3=&s=Roma), [Eternal City#1](http://wordnetweb.princeton.edu/perl/webwn?o2=&o0=1&o7=1&o5=&o1=1&o6=&o4=&o3=&s=Eternal+City), [Italian capital#1](http://wordnetweb.princeton.edu/perl/webwn?o2=&o0=1&o7=1&o5=&o1=1&o6=&o4=&o3=&s=Italian+capital), [capital of Italy#1](http://wordnetweb.princeton.edu/perl/webwn?o2=&o0=1&o7=1&o5=&o1=1&o6=&o4=&o3=&s=capital+of+Italy) (capital and largest city of Italy; on the Tiber; seat of the Roman Catholic Church; formerly the capital of the Roman Republic and the Roman Empire)

(n) **Rome#2** (the leadership of the Roman Catholic Church)

[**Warsaw**](http://wordnetweb.princeton.edu/perl/webwn?o2=&o0=1&o7=1&o5=&o1=1&o6=&o4=&o3=&s=Warsaw&i=0&h=0#c)

(n) [Warszawa#1](http://wordnetweb.princeton.edu/perl/webwn?o2=&o0=1&o7=1&o5=&o1=1&o6=&o4=&o3=&s=Warszawa), **Warsaw#1**, [capital of Poland#1](http://wordnetweb.princeton.edu/perl/webwn?o2=&o0=1&o7=1&o5=&o1=1&o6=&o4=&o3=&s=capital+of+Poland) (the capital and largest city of Poland; located in central Poland)

**north**

(n) **north#3**, due north#1, northward#1, N#2 (the cardinal compass point that is at 0 or 360 degrees)

(n) **north#4** (a location in the northern part of a country, region, or city)

(n) **north#5** (the direction corresponding to the northward cardinal compass point)

(n) **North#1** (the region of the United States lying to the north of the Mason-Dixon line)

(n) Union#2, **North#2** (the United States (especially the northern states during the American Civil War))

(n) **north#6**, magnetic north#1, compass north#1 (the direction in which a compass needle points)

(n) **North#7**, Frederick North#1, Second Earl of Guilford#1 (British statesman under George III whose policies led to rebellion in the American colonies (1732-1792))

**south**

(n) **south#3**, due south#1, southward#1, S#3 (the cardinal compass point that is at 180 degrees)

(n) **south#4** (a location in the southern part of a country, region, or city)

(n) **south#5** (the direction corresponding to the southward cardinal compass point)

(n) **South#1** (the region of the United States lying to the south of the Mason-Dixon line)

(n) Confederacy#1, Confederate States#1, Confederate States of America#1, **South#2**, Dixie#1, Dixieland#1 (the southern states that seceded from the United States in 1861)

**east**

(n) **east#1**, due east#1, eastward#1, E#3 (the cardinal compass point that is at 90 degrees)

(n) **east#4** (the direction corresponding to the eastward cardinal compass point)

(n) **east#5** (a location in the eastern part of a country, region, or city)

(n) **East#2**, Orient#1 (the countries of Asia)

(n) **East#3**, eastern United States#1 (the region of the United States lying to the north of the Ohio River and to the east of the Mississippi River)

**west**

(n) **west#2**, due west#1, westward#1, W#2 (the cardinal compass point that is a 270 degrees)

(n) **west#4** (the direction corresponding to the westward cardinal compass point)

(n) **west#8** (a location in the western part of a country, region, or city)

(n) **West#1**, Occident#1 (the countries of (originally) Europe and (now including) North America and South America)

(n) **West#3**, western United States#1 (the region of the United States lying to the west of the Mississippi River)

(n) **West#5**, Rebecca West#1, Dame Rebecca West#1, Cicily Isabel Fairfield#1 (British writer (born in Ireland) (1892-1983))

(n) **West#6**, Mae West#1 (United States film actress (1892-1980))

(n) **West#7**, Benjamin West#1 (English painter (born in America) who became the second president of the Royal Academy (1738-1820))

|  |  |
| --- | --- |

**Germany**

(n) **Germany#1**, Federal Republic of Germany#2, Deutschland#1, FRG#1 (a republic in central Europe; split into East Germany and West Germany after World War II and reunited in 1990)

**Poland**

(n) **Poland#1**, Republic of Poland#1, Polska#1 (a republic in central Europe; the invasion of Poland by Germany in 1939 started World War II)

**Russia**

(n) Soviet Union#1, **Russia#1**, Union of Soviet Socialist Republics#1, USSR#1 (a former communist country in eastern Europe and northern Asia; established in 1922; included Russia and 14 other soviet socialist republics (Ukraine and Byelorussia and others); officially dissolved 31 December 1991)

(n) Soviet Russia#1, **Russia#2**, Russian Soviet Federated Socialist Republic#1 (formerly the largest Soviet Socialist Republic in the USSR occupying eastern Europe and northern Asia)

(n) **Russia#3** (a former empire in eastern Europe and northern Asia created in the 14th century with Moscow as the capital; powerful in the 17th and 18th centuries under Peter the Great and Catherine the Great when Saint Petersburg was the capital; overthrown by revolution in 1917)

(n) **Russia#4**, Russian Federation#1 (a federation in northeastern Europe and northern Asia; formerly Soviet Russia; since 1991 an independent state)

**Greece**

(n) **Greece#1**, [Hellenic Republic#1](http://wordnetweb.princeton.edu/perl/webwn?o2=&o0=1&o7=1&o5=&o1=1&o6=&o4=&o3=&s=Hellenic+Republic), [Ellas#1](http://wordnetweb.princeton.edu/perl/webwn?o2=&o0=1&o7=1&o5=&o1=1&o6=&o4=&o3=&s=Ellas) (a republic in southeastern Europe on the southern part of the Balkan peninsula; known for grapes and olives and olive oil)

(n) **Greece#2** (ancient Greece; a country of city-states (especially Athens and Sparta) that reached its peak in the fifth century BCE)

**France**

(n) **France#1**, [French Republic#1](http://wordnetweb.princeton.edu/perl/webwn?o2=&o0=1&o7=1&o5=&o1=1&o6=&o4=&o3=&s=French+Republic) (a republic in western Europe; the largest country wholly in Europe)

(n) **France#2**, [Anatole France#1](http://wordnetweb.princeton.edu/perl/webwn?o2=&o0=1&o7=1&o5=&o1=1&o6=&o4=&o3=&s=Anatole+France), [Jacques Anatole Francois Thibault#1](http://wordnetweb.princeton.edu/perl/webwn?o2=&o0=1&o7=1&o5=&o1=1&o6=&o4=&o3=&s=Jacques+Anatole+Francois+Thibault) (French writer of sophisticated novels and short stories (1844-1924))

**Italy**

(n) **Italy#1**, [Italian Republic#1](http://wordnetweb.princeton.edu/perl/webwn?o2=&o0=1&o7=1&o5=&o1=1&o6=&o4=&o3=&s=Italian+Republic), [Italia#1](http://wordnetweb.princeton.edu/perl/webwn?o2=&o0=1&o7=1&o5=&o1=1&o6=&o4=&o3=&s=Italia) (a republic in southern Europe on the Italian Peninsula; was the core of the Roman Republic and the Roman Empire between the 4th century BC and the 5th century AD)

[**Austria**](http://wordnetweb.princeton.edu/perl/webwn?o2=&o0=1&o7=1&o5=&o1=1&o6=&o4=&o3=&s=austria&i=0&h=0#c)

(n) **Austria#1**, [Republic of Austria#1](http://wordnetweb.princeton.edu/perl/webwn?o2=&o0=1&o7=1&o5=&o1=1&o6=&o4=&o3=&s=Republic+of+Austria), [Oesterreich#1](http://wordnetweb.princeton.edu/perl/webwn?o2=&o0=1&o7=1&o5=&o1=1&o6=&o4=&o3=&s=Oesterreich) (a mountainous republic in central Europe; under the Habsburgs (1278-1918) Austria maintained control of the Holy Roman Empire and was a leader in European politics until the 19th century)

[**Spain**](http://wordnetweb.princeton.edu/perl/webwn?o2=&o0=1&o7=1&o5=&o1=1&o6=&o4=&o3=&s=Spain&i=0&h=0#c)

(n) **Spain#1**, [Kingdom of Spain#1](http://wordnetweb.princeton.edu/perl/webwn?o2=&o0=1&o7=1&o5=&o1=1&o6=&o4=&o3=&s=Kingdom+of+Spain), [Espana#1](http://wordnetweb.princeton.edu/perl/webwn?o2=&o0=1&o7=1&o5=&o1=1&o6=&o4=&o3=&s=Espana) (a parliamentary monarchy in southwestern Europe on the Iberian Peninsula; a former colonial power)
