# Supplementary material for: Structural Similarities between Brain and Linguistic Data Provide Evidence of Semantic Relations in the Brain
Source: PLoS One. 2013 Jun 14;8(6):e65366. doi: 10.1371/journal.pone.0065366 (PMC3682999; doi:10.1371/journal.pone.0065366)
Supplement: Material S3 — Five Wordnet-based measures of similarity. (DOCX) [file pone.0065366.s004.docx]

## S3 -- Five Wordnet-based measures of similarity

By default, the similarity measure for a word with itself is 1. For all other word and word sense pairs, the average of the following five measures were taken. The first measure, wup, due to (Wu and Palmer, 1994) is a similarity measure based on path lengths between concepts. The wup measure uses the depth of each concept, that is, the length of the path from the root node to the synset, and the depth of the least common subsumer (LCS) of the two concepts, which is the most specific concept they share as an ancestor. The formula for synsets s1 and s2 is wup(s1,s2) = 2 * depth(LCS(s1,s2)) / (depth(s1) + depth(s2)). If the two synsets are the same the score is 1. The score can never be 0 because the depth of the LCS is never 0 (the depth of the root is 1). 0<x<=1

The two similarity measures of lin (Lin 1998) and jcn (Jiang and Conrath 1997) are based on the notion of information content (IC). IC is a corpus–based measure of the specificity of a concept, measured in terms of the frequency of occurrence of the concept in the corpus. The more specific the concept, the lower its frequency and the higher its IC score. By default, the information content of concepts in these measures is derived from the human-annotated, sense–tagged corpus SemCor (Miller *et al*., 1993). This semantic concordance links every word in the Brown Corpus to its appropriate WordNet sense. The lin measure scales the information content of the LCS by the sum of the information content of the individual concepts. The jcn measure subtracts the information content of the LCS from this sum and takes the inverse to convert the distance to a similarity measure.

lin(s1,s2) = 2 * IC(LCS(s1,s2)) / (IC(s1) + IC(s2))

jcn(s1,s2) = 1 / (IC(s1) + IC(s2) - 2 * IC(LCS(s1,s2)))

In using information content of the least common subsumer (LCS) of the two concepts, lin and jcn also take some account of path length. The lin similarity score is always greater than or equal to 0 and less than or equal to 1. If the information content of s1 and s2 are 0, 0 is returned as the score. Similarly for jcn. If IC(s1) + IC(s2) = 0, a score of 0 is returned. If IC(s1) + IC(s2) = 2 * IC(LCS(s1,s2)), which would be the case if the two input synsets were the same, a maximum relatedness score is returned by finding the smallest possible distance greater than 0 and returning the multiplicative inverse of that distance.

The last two measures of similarity rely on vector-space models (Banerjee and Pedersen, 2002; Pedesen et al., 2004). The Gloss Vector (gv) measure works by forming second-order co-occurrence vectors from the WordNet definitions of concepts, known as glosses. It augments the gloss of each synset with the glosses of adjacent concepts as defined by WordNet relations. The Pairwise Gloss Vector (pgv) measure augments the glosses by forming separate vectors for the hyponyms, holonyms, meronyms, and so on instead of forming a single combined vector for each concept. These scores are also all between 0 and 1.

Banerjee S, Pedersen T (2002) An Adapted Lesk Algorithm for Word Sense Disambiguation using WordNet. In Proceedings of the Third International Conference on Intelligent Text Processing and Computational Linguistics, pp. 136-145, February 17-23, 2002, Mexico City.

Jiang J, Conrath D (1997) Semantic similarity based on corpus statistics and lexical taxonomy. In *Proceedings of International Conference on Research in Computational Linguistics,* Taiwan. 19-33.

Leacock C, Chodorow M (1998) Combining local context and WordNet similarity for word sense identification. In Fellbaum (Ed.) *WordNet: An Electronic Lexical Database*. Cambridge, MA: MIT Press. pp. 265-283.

Lin D (1998) An information-theoretic definition of similarity. In *Proc. 15th International Conf. on Machine Learning*. Morgan Kaufmann, San Francisco, CA, p. 296--304.

Miller GA, Leacock C, Tengi R, Bunker RT (1993) [A Semantic Concordance.](http://acl.ldc.upenn.edu/H/H93/H93-1061.pdf) In: *Proceedings of the 3 DARPA Workshop on Human Language Technology*.

Pedersen T, Patwardhan S, Michelizzi, J (2004) WordNet::Similarity - Measuring the Relatedness of Concepts. In: *Proceedings of Fifth Annual Meeting of the North American Chapter of the Association for Computational Linguistics (NAACL-2004)* , pp. 38-41. Boston, May 2004.

Wu Z, Palmer M (1994) Verb Semantics and Lexical Selection. In *Proceedings of the 32nd Annual Meeting of the Association for Computational Linguistics*. Las Cruces, New Mexico. pp. 133--138 .
